# Enhancing the network specific individual characteristics in rs‐fMRI functional connectivity by dictionary learning

**DOI:** 10.1002/hbm.26289

**Published:** 2023-04-18

**Authors:** Pratik Jain, Ankit Chakraborty, Rakibul Hafiz, Anil K. Sao, Bharat Biswal

**Affiliations:** ^1^ School of Computing and Electrical Engineering Indian Institute of Technology Mandi Mandi India; ^2^ Department of Biomedical Engineering New Jersey Institute of Technology Newark New Jersey 07102 USA; ^3^ Department of Electrical Engineering and Computer Science Indian Institute of Technology Bhilai Bhilai India

**Keywords:** brain atlas, degree normalization, dictionary learning, Fisher Z transform, fMRI, functional connectivity, individual connectome, resting‐state networks

## Abstract

Most fMRI inferences are based on analyzing the scans of a cohort. Thus, the individual variability of a subject is often overlooked in these studies. Recently, there has been a growing interest in individual differences in brain connectivity also known as individual connectome. Various studies have demonstrated the individual specific component of functional connectivity (FC), which has enormous potential to identify participants across consecutive testing sessions. Many machine learning and dictionary learning‐based approaches have been used to extract these subject‐specific components either from the blood oxygen level dependent (BOLD) signal or from the FC. In addition, several studies have reported that some resting‐state networks have more individual‐specific information than others. This study compares four different dictionary‐learning algorithms that compute the individual variability from the network‐specific FC computed from resting‐state functional Magnetic Resonance Imaging (rs‐fMRI) data having 10 scans per subject. The study also compares the effect of two FC normalization techniques, namely, Fisher Z normalization and degree normalization on the extracted subject‐specific components. To quantitatively evaluate the extracted subject‐specific component, a metric named Overlap is proposed, and it is used in combination with the existing differential identifiability $\left(I_{\text{diff}}\right)$ metric. It is based on the hypothesis that the subject‐specific FC vectors should be similar within the same subject and different across different subjects. Results indicate that Fisher Z transformed subject‐specific fronto‐parietal and default mode network extracted using Common Orthogonal Basis Extraction (COBE) dictionary learning have the best features to identify a participant.

## Graphical abstract

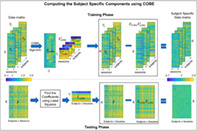

## 1 INTRODUCTION

Functional magnetic resonance imaging (fMRI) is a popular noninvasive neuroimaging technique that accommodates high spatial and temporal resolution to perform systems‐level neuroscience in human and animal models. Currently, most fMRI studies use the Blood oxygenation level‐dependent (BOLD) contrast mechanism first proposed by Ogawa et al. (1990). Ogawa and colleagues showed that changes in blood oxygen levels in the brain (oxygenated blood) can be reflective of the neuronal response during some cognitive or mental task. Since fMRI signals are “dependent” and sensitive to these changes, they are called “Blood Oxygenation Level Dependent” (BOLD) signals. Generally, for task activation studies, subjects are presented with a stimulus for a short period (10–20 s), alternating with about the same period of a control condition. Although task‐fMRI has been widely used to identify brain regions corresponding to specific tasks, it is still not clear which stimulus or task could be appropriate for finding out the unique characteristics of the individual which make every person different. In addition, certain populations such as infants, Alzheimer's patients, and patients with other debilitating clinical disorders may not be able to perform certain tasks required from them.

Resting‐state fMRI (rs‐fMRI) has emerged as an alternative to task‐fMRI to map brain functions by observing brain signals during rest. This method was first demonstrated in 1995, where it was shown that brain activations in the resting‐state could exhibit similar correlations between brain regions as activations in the task‐state (Biswal et al., 1995). rs‐fMRI primarily focuses on measuring the spontaneous activity in BOLD signals, which is measured in a resting state wherein subjects do not perform specific tasks that may alter brain activity. These rs‐fMRI signals possess very low amplitude fluctuations, resting primarily within the 0.01–0.1 Hz range (van den Heuvel & Hulshoff Pol, 2010). It was shown that rs‐fMRI signals in the sensorimotor and its associated cortex had a significant temporal correlation within the cortex but not with other brain regions. Similar observations were also made in other functional regions, including the visual cortex (Lowe et al., 1998).

Friston et al. (1993) first defined functional connectivity (FC) in neuroimaging as temporal correlations between spatially remote neurophysiological events. Because we observed temporal correlation between functionally related regions during resting state, specifically, a high temporal correlation between the fMRI time series between the left and right primary motor network, we used the term resting state functional connectivity (RSFC). These left and right hemispheric regions are spatially distributed but seem to be functionally connected and share information with each other. Various studies have demonstrated similar significant correlations in the other known networks including visual network, auditory network, and other cognitive networks (Biswal et al., 1997; Cordes et al., 2000, 2002; Damoiseaux et al., 2006; De Luca et al., 2005; Fox & Raichle, 2007; Greicius et al., 2003; Lowe et al., 2000; van den Heuvel et al., 2008; Xiong et al., 1999).

In general, FC from rs‐fMRI can be computed using two widely used methods. The first is data‐driven approaches such as independent component analysis (ICA) that decomposes the fMRI data into spatially segregated components based on how well the voxels within a component are temporally synchronized. The other method is the seed‐based correlation method. It performs the temporal correlation between fMRI time series voxels. The seed‐based method needs prior knowledge of the brain regions of interest (ROIs). Pairwise correlation can then be performed between all ROI's time series to generate a functional brain network.

Rs‐fMRI possesses several advantages over task‐based fMRI. For one, it is a simpler paradigm that does not require stimuli to be presented to a subject, nor does it require the subject to respond to stimuli. It is also easier for certain patient groups, such as the very young or elderly, to undergo imaging as they do not need to perform actions they may have difficulty with (Maknojia et al., 2019). Additionally, it has been found that rs‐fMRI can pick up on trends in the brain that task‐based fMRI cannot pick up on as well. For example, one study has used rs‐fMRI to classify the social and neurocognitive performance in individuals based on connectivity in sensorimotor networks. Task‐based fMRI was also used for this study but was less sensitive to detecting connectivity in the brain, and its findings did not replicate well across another independent test sample, whereas rs‐fMRI did (Viviano et al., 2019). Besides, Finn et al. (2017) compared the unique individual characteristics from the Human Connectome Project (HCP) dataset, which contains two resting‐state and seven different task‐based fMRI scans for each subject. The study correlated the FC computed from every pair of fMRI scans for the same subject. It found out that the correlation between the FCs of the two resting‐state scans was higher than the other rest–task or task–task pairs. However, the rest–rest correlation decreased when they reduced the number of time points while calculating the FC, suggesting that more time points are essential for computing the FC.

Recently, there has been a growing interest in individual differences in Brain Connectivity also known as Individual Connectome. Unlike structural MRI, which is widely used in clinical applications to describe the physical structure of individual brains, fMRI, and RSFC have faced a lot of challenges in extracting the individual‐specific information limiting its clinical use (Gordon et al., 2017). Individual Connectome can help us understand and predict the behavior right from the RSFC as demonstrated by Finn et al. (2015) which further helps in creating biomarkers or fingerprints that can identify an individual from a cohort. This can also be used as a brain‐based measure on an individual brain.

One aspect of quantifying the individual differences has been to predict the behavioral scores from the resting state brain scans. Earlier, the neural correlates of individual differences in general fluid intelligence (Gf) have been found to be associated with variations in brain size and connections (Deary, 2012; Jaeggi et al., 2008). Recently, the information flow among certain areas associated with Gf has been quantified by FC. These results suggest that the variation in the correlation between specific brain regions is related to specific behavioral factors that may contribute to individual differences (Haier, 2009; Penke et al., 2012; Song et al., 2008). The whole‐brain FC measure may provide a more holistic sight to determine an individual's Gf rather than global brain size. A recent study Li et al. (2020) used, a general linear model to predict the Gf scores from healthy participants (*N* = 326) using their FCNs. Moreover, the results of the model were validated by a leave‐one‐out cross‐validation approach, and its performance was measured by computing Pearson's correlation between the predicted and actual Gf scores. The model found FCs in the superior longitudinal fasciculus, deep frontal regions, and ventral fronto‐parietal important for Gf prediction. This result demonstrates that FCs can be used as a predictor of Gf and can potentially be used to predict other phenotypic traits. Furthermore, researchers have reported that the individual connectome can improve prediction performance (Cai et al., 2019; Kashyap et al., 2019; Qin et al., 2019). Thus, studies on extracting the subject‐specific FC have come into prominence.

Measuring and validating the subject‐specific components can be subjective. Generally, the validation of the subject‐specific components is based on the assumption that the connectivity profiles should be more similar between visits of the same subjects than between different subjects. Finn et al. (2017) have attempted to explain this assumption using a thought experiment considering Human faces and used the term identifiability as a metric to evaluate the subject‐specific component. They argue that there can be two ways to enhance the identifiability: first by exaggerating the most prominent features of each individual, which make individuals look more different from each other, and second by removing the irrelevant or redundant features while retaining and enriching relevant ones. Moreover, Amico and Goñi (2018) uses PCA to remove the redundant features and demonstrates that this can significantly enhance the identifiability. They defined the term differential identifiability $I_{\text{diff}}$, which quantifies the difference between the average within‐subject FCs similarity and the average between subjects FCs similarity. Most of the studies try to predict the behavioral scores of an individual from the subject‐specific information computed from the FC of the same individual.

Several studies have attempted to predict the behavioral scores from RSFC using different machine‐learning techniques. Kashyap et al. (2019) showed how the prediction of behavioral scores could be enhanced by first removing the common components in the RSFC using Common Orthogonal Basis Extraction (COBE) method. Recent work has used more complex methods such as Multi‐Task Learning‐based sparse Convex Alternating Structure Optimization (MTL‐sCASO; Wang et al., 2021) to construct the RSFC in a regularized learning framework. The MTL‐sCASO method defines a term to encompass the effect of the trade‐off between the similar network topology generally shared among individuals and the inter‐individual variability in estimating the brain network during rest. On the other hand, there are studies that have focused on a specific attribute such as, attention (Rosenberg et al., 2016), gender (Smith et al., 2014), procrastination (Wu et al., 2016), age (Geerligs et al., 2015), along with intelligence (Levakov et al., 2021) finding individual differences in bilingual individuals (Nichols et al., 2021), and predicting individual differences in propensity to trust (Lu et al., 2019).

We can estimate subject‐specific components from either the fMRI BOLD signal time series or from the corresponding FC networks. Extracting the subject‐specific components from the BOLD signal during a task stimulus is preferred as all the subjects are presented with the same stimulus representing similar information of the subject's response. While during resting‐state it is highly probable that the BOLD signal of every subject is different as per their thoughts during the rs‐fMRI scan. Yet, it is shown that the RSFC networks are consistent among the subjects during the rs‐fMRI scan making FC a better choice over the BOLD signal to get the subject‐specific components. Iqbal et al. (2018) demonstrated how the shared and the subject‐specific components could be extracted from the BOLD signal of each voxel in task fMRI using the shared and subject‐specific dictionary learning (ShSSDL) algorithm. The ShSSDL algorithm generates a group level as well as a set of subject‐specific voxel‐wise spatial maps by simultaneously learning multiple dictionaries about the analyzed task fMRI datasets. While the ShSSDL is performed voxel‐wise, Kashyap et al. (2019) have used the COBE algorithm that uses ROI‐based time‐courses. They used 400 parcellations by Schaefer et al. (2018) on (Yeo et al., 2011) brain atlas, which results in shared and individual time series that is further used to compute the FC from resting‐state data.

Several studies have attempted to extract individual differences at the FC level. Researchers have used similarity measures such as Pearson correlation between FCs computed by two or more scans of the same subject (Finn et al., 2015, 2017; Kraus et al., 2021) others compute them with algorithms that either exploit the sparse nature of the FC (Wang et al., 2021) or use analysis techniques like representational similarity analysis (Nichols et al., 2021). There are other studies that use machine learning‐based methods like Amico and Goñi (2018) that demonstrate how principal component analysis (PCA) can enhance the individual differences and Qin et al. (2019) showed how low‐rank learning algorithms like Robust PCA (RPCA) could also improve these differences. Moreover, K‐SVD, a well‐known algorithm in machine learning, was used by Cai et al. (2019) which shows encouraging results. On the other hand, Pallarés et al. (2018) have attempted to bring out the individual differences from effective connectivity (EC), which uses causality to compute the connectivity matrix, unlike FC, which uses Pearson correlation.

A vital requirement for estimation of FC is to choose a suitable Brain Atlas from the many available in the literature. Finn et al. (2015) initially demonstrated results with the functional atlas developed by Shen et al. (2013) having 268 ROIs and also compared it with Free Surfer atlas (Fischl et al., 2004) having 68 ROIs, concluding higher resolution atlas boosts identification rate within the subjects. Wang et al. (2021) have also compared different parcellation schemes and have emphasized on using Whole Brain atlas including the subcortical regions. Cai et al. (2020), Lu et al. (2019), Rosenberg et al. (2016), and Shen et al. (2017) have also used the Shen 268 node atlas, while studies like Qin et al. (2019) have used the Dosenbach 160 node (Dosenbach et al., 2010) atlas, and other studies Kashyap et al. (2019), Levakov et al. (2021), and Pessoa et al. (2021) have used the Schaefer atlas. Recently, Kong et al. (2019) proposed a multi‐session hierarchical Bayesian Model (MS‐HBM) to estimate functional network parcellations of the cerebral cortex in individual subjects. MS‐HBM model attempts to estimate individual‐specific cerebral cortex parcellations using multi‐session fMRI data. Thus, many studies have attempted to use different brain atlases throughout the literature, but very few have compared the effect of varying the atlas.

Normalization of FC is something that is not very well reported in the literature, various studies like Finn et al. (2017), Geerligs et al. (2015), Gordon et al. (2017), and Kraus et al. (2021) have used the Fisher Z normalization over the FC, while other studies like Amico and Goñi (2018), Cai et al. (2019), and Kashyap et al. (2019) have directly used FC without Fisher Z normalization. However, a recent study Chiem et al. (2021) reported that by using degree normalization one could improve the differential identifiability $I_{\text{diff}}$ score. This shows that normalization is essential, and hence in this study, we also study the effect of normalization on the subject‐specific component.

In this article, we reviewed four dictionary learning (DL) methods that have been used to determine individual‐specific connectivity or components, that is, PCA, RPCA, KSVD, and COBE. We use two publicly available datasets that have multiple sessions of scanning data sets. In the first data set, the Midnight Scan Club (MSC) had scanned 10 subjects with 10 sessions per subject. For the second data set, HNU, had scanned 30 subjects with 10 sessions per subject. Each of these datasets was split into training and testing sets, the dictionaries were learned using the training set and they were further used to decompose the test set to get the subject‐specific component. This analysis was performed on the FC computed by each of the seven resting‐state networks (RSNs) given by Yeo et al. (2011). We compare the different RSNs in terms of which network has the maximum subject‐specific information. We have used a grid search to determine the values of the hyper‐parameters involved in each of the DL algorithms. Further, we have checked the reproducibility and generalizability by repeating the analysis each time choosing a different set of data to train the dictionaries of the DL algorithms. We also report the effect of varying the time‐points and the effect of changing the parcellation scheme (Brain atlas) which in turn affects the FC computation. Normalization methods have also been taken into consideration by repeating the analysis first without any normalization method and then by Fisher Z and degree normalization.

## 2 MATERIALS AND METHODS

### 2.1 Resting‐state fMRI data

This study employed two publicly available datasets: Hangzhou Normal University (HNU) and Midnight Scan Club (MSC).

#### 2.1.1 The MSC datasets

The MSC dataset includes 10 healthy individuals (5 males, 5 females) aged between 24 and 31 years of age. The scanning protocol was approved by the local Institutional Review Board at Washington University in St. Louis. All subjects signed informed consent. Each subject was scanned for four T1 weighted images with a Siemens Trio Tim scanner with repetition time (TR) = 2400 ms, echo time (TE) = 3.74 ms, flip angle = 8°, voxel size of $0.8\times 0.8\times 0.8\;mm^{3}$ having 224 Sagittal slices. Along with this, each subject underwent 10 rs‐fMRI scans each half an hour long with TR = 2200 ms, TE = 27 ms, flip angle = 90°, and 36 axial slices with 818 volumes. Subjects were instructed to keep their eyes open while observing a plus sign. More details can be found in Gordon et al. (2017).

#### 2.1.2 HNU dataset

The HNU dataset includes 30 healthy individuals (15 males, 15 females) aged between 20 and 30 years of age. Each participant was scanned 10 times in 1 month, with each scanning occurring every 3 days. The study was approved by the local IRB and informed consent was obtained from all the subjects prior to scanning. All the data was collected using a 3T GE Scanner. The anatomical T1 weighted images were acquired using a 3D SPGR sequence with TR 8.06 ms, flip angle of 8°, voxel size of $1\times 1\times 1\;mm^{3}$ with 180 Sagittal slices. Scan parameters for the EPI sequence used to collect the rs‐fMRI data include TR = 2000 ms, TE = 30 ms, flip angle = 90°, 43 axial slices with image size 64×64, voxel size of $3.4\times 3.4\times 3.4\;mm^{3}$. Each resting‐state scan lasted for 10 min which resulted in 300 volumes. During the rs‐fMRI data collection, participants were asked to keep their eyes open while observing a fixation cross. More details can be found in Chen et al. (2015) and Zuo et al. (2014).

### 2.2 Data preprocessing

**FIGURE 1:**
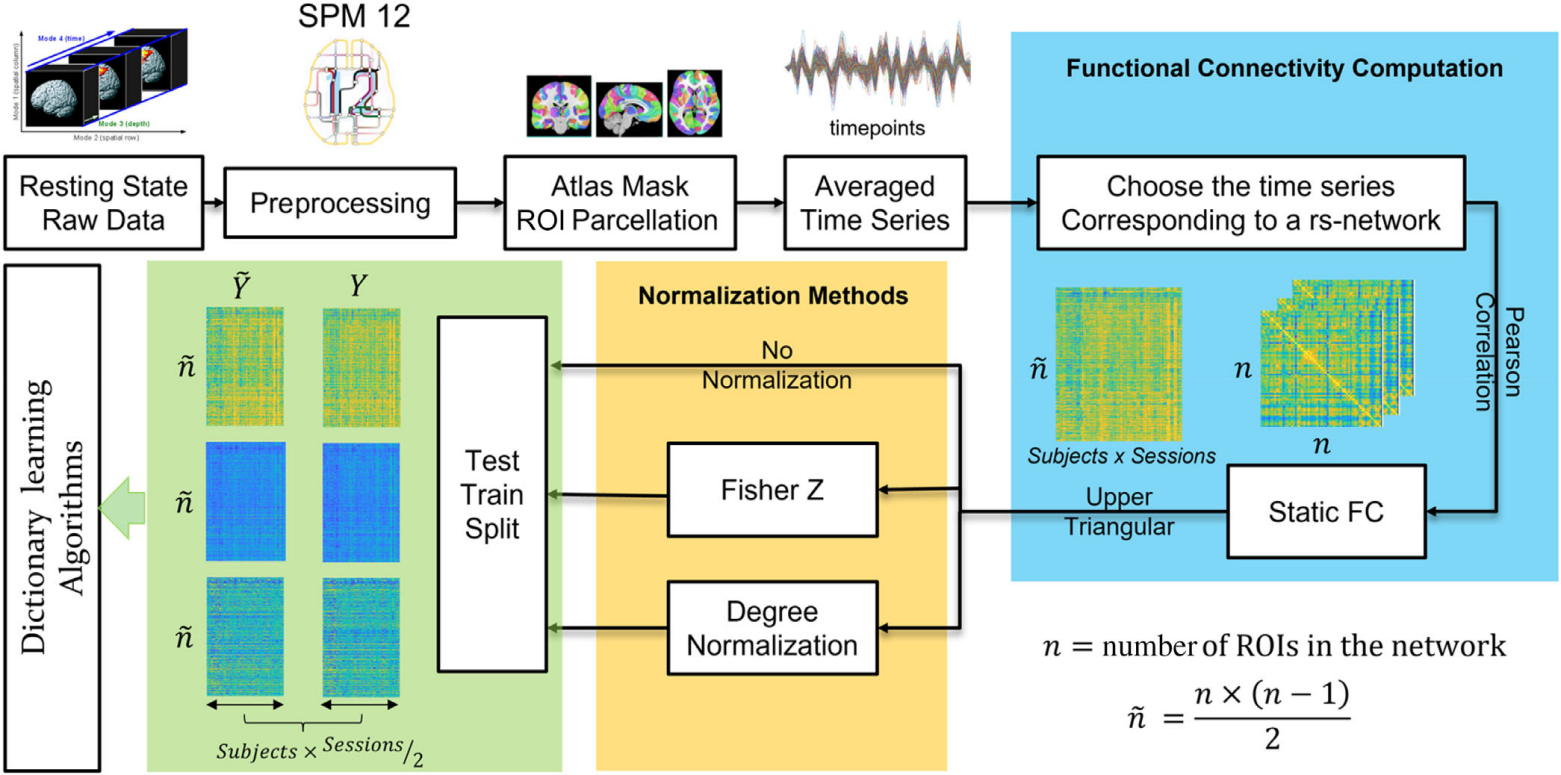
Overview of preprocessing, FC computation, and the Train‐Test Split. The 4‐dimensional rs‐fMRI data are preprocessed in MATLAB 2019b using the SPM 12 toolbox, further segmented into ROIs using a predefined Brain Atlas. The BOLD time courses within an ROI are averaged to get one time course for an ROI. Moreover, the time courses belonging to each of the seven networks (Yeo et al., 2011) are chosen one by one, and an FC matrix specific to the rs‐network is formed using Pearson Correlation. Since this correlation matrix is symmetric, only the upper‐triangular elements are vectorized, and a data matrix is formed by concatenating the upper triangular vectors from each scan. The train‐test split is further performed by placing five sessions per subject for training and the other five for test over the data matrix, the Fisher Z transformed, and the Degree Normalized data matrix. These matrices are given as input to the dictionary learning algorithms.

The data from both sites were preprocessed using the same pipeline. We preprocessed the raw rsfMRI data with the SPM12 toolbox (http://www.fil.ion.ucl.ac.uk/spm/). First, we discarded the first 10 volumes for the MSC data for T1 saturation effects. For the HNU data set, the first 50 data points were excluded to further avoid susceptibility issues. Motion correction algorithms were performed for the detection and correction of head motion‐related signal changes. Briefly, the preprocessing included five steps: slice timing correction, realignment, coregistration, normalization, and smoothing. Slice time correction corrects the differences in image acquisition time between the slices. Realignment realigns the time‐series of images acquired from the same scan using a least‐squares approach and a six parameter (rigid body) spatial transformation. Motion correction is done in this stage. The T1‐weighted anatomical image of each subject was coregistered to the corresponding functional scans. Normalization was performed to nonlinearly transform the functional data to the standard Montreal Neurological Institute (MNI) space. The spatial resolution of each voxel was resampled to isotropic $2\times 2\times 2\;mm^{3}$. The normalized functional images were then smoothed with a Gaussian kernel of 8 mm. The time‐series signal from each voxel was then filtered in the frequency range of 0.01–0.1 Hz. Figure 1 shows the Overview of the preprocessing, Atlas parcellation, FC computation, normalization, and making the training and test matrix which will be given as input to the DL algorithms.

### 2.3 Brain atlas

Parcellation divides each cerebral hemisphere into anatomical and/or functional ROIs. To identify different functional brain regions, standard brain atlas commonly used in fMRI literature were used. For this study, Schaffer 400 atlas (Schaefer et al., 2018) was used. Briefly, it used both task and resting‐state fMRI to generate a gradient‐weighted Markov Random Field (gwMRF) model integrating local gradient and global similarity approaches. Applying the gwMRF model on about 1500 subjects, Schaefer and colleagues could generate atlases with different numbers of ROIs such as 100, 200, 300, up to 1000, distributed across several functional networks. The preprocessed data were parcellated using nine different predefined brain atlases of different resolutions to show the effect of the Brain atlas on the estimated individual components. Several complimentary atlases, which were publicly available and commonly used, such as Dosenbach, Brainnetome, Power, Shen, and Seitzman were also included in the study. Dosenbach atlas was created by several meta‐analyses of task‐fMRI activation studies. Brainnetome atlas was created by initially starting with an automatic surface parcellation and subcortical segmentation using free surfer's Desikan–Killiany atlas (Desikan et al., 2006) and modifying it with the structural information from diffusion tensor imaging (DTI) and functional information from RSFC acquired from 40 subjects. The Power atlas used a combination of task fMRI and rs‐fMRI to create 264 nodes in the brain atlas. Brain regions that reliably displayed significant activity when certain tasks such as button pressing were performed identified around 151 ROIs. Further FC mapping techniques were used from rs‐fMRI data of 40 subjects to define the other 193 ROIs. The Shen atlas was created using a Groupwise multigraph K‐way spectral clustering algorithm that jointly optimizes the group and the individual parcellation. Last, Seitzman and colleagues modified the power atlas using a winner take all partitioning technique to make the Seitzman atlas.

Table 1 gives the summary of the atlases used in this study. We have also reported the average number of voxels per ROI for every atlas used in this study, which is crucial in selecting the atlas. We have included atlases such as Dosenbach (Dosenbach et al., 2010), Power (Power et al., 2011), and Seitzman atlas (Seitzman et al., 2020), that do not assign every voxel in the brain to a region, instead they define a spherical region around a seed voxel. Figure S6 shows the atlas views of all the atlases used in this study. Further, for a fair comparison of RSNs present in each of the atlases, the Yeo 7 resting‐state (RS) network atlas (Yeo et al., 2011) was overlaid on each of these atlases, and the nodes of the atlas were assigned a particular Yeo network by checking the extent of overlap of each of the Yeo networks with the node. The network which was maximally overlapped with the node was assigned to the node. A similar approach has been used by van Geest et al. (2018).

**TABLE 1:** Brief details of atlases.

Atlas	ROIs	Method	Average number of voxels per ROI	References
Dosenbach	160	Meta‐analysis of task‐fMRI activation studies.	123	(Dosenbach et al., 2010)
Brainnetome	246	Structural and functional information.	477	(Fan et al., 2016)
Power	264	Used a combination of task based and rs‐fMRI data.	8	(Power et al., 2011)
Shen	268	Groupwise multigraph K‐way spectral clustering algorithm.	601	(Shen et al., 2013)
Seitzman	300	Winner takes all partitioning technique.	58	(Seitzman et al., 2020)
Schaefer	100, 200, 300, 400	gwMRF.	1320, 660, 440, 330	(Schaefer et al., 2018)

### 2.4 FC computation

Here, we briefly describe the FC computation done in this study. Instead of calculating the FC of the whole brain, we have calculated the network‐specific FC for each of the 7 Yeo rs‐networks. After identifying the preprocessed data with the different atlas mentioned before, the BOLD time series corresponding to each ROI were averaged, and a mean time series was formed. A pairwise correlation was performed between averaged time series from each region, which results in n×n correlation matrix, where *n* is the number of ROIs in the atlas corresponding to an RS network. The resultant correlation matrix is symmetric and transformed into a vector, defined as an FC vector, by considering only the upper triangular part of the FC matrix. The length of the FC vector is $\tilde{n}=\frac{n\left(n-1\right)}{2}$. We have 10 such FC vectors for an RS‐network per subject since we have 10 sessions per subject. These correlation values are also normalized using Fisher Z transformation and degree normalization to report the effects of normalization methods. The details of Fisher Z transformation and degree normalization are given below.

### 2.5 Normalizing the FC

The FC matrix is the output of Pearson Correlation and hence is in the range from [−1, 1]. Since these are correlation values, we could use some well‐defined transformations like the Fisher Z transformation that works on the distribution of the correlation values. Fisher Z transforms as discussed below is a transform that works on every correlation value independent of the other. On the other hand, techniques like degree normalization treat the FC matrix as an adjacency matrix and scale every correlation value according to the values that are in its neighborhood.

#### 2.5.1 Fisher Z

The distribution of correlation value for a given RS‐network is highly skewed (Ehtemami et al., 2018; Silver & Dunlap, 1987), hence a normalizing transform developed by Fisher is used for converting the skewed distribution of the sample correlation into a normal distribution. The Fisher's Z transformation is given by,

(1) $$z=0.5\;\left[\ln\left(1+r\right)-\ln\left(1-r\right)\right].$$

Here, r refers to the Pearson correlation value and z refers to the corresponding Fisher Z transformation. Fisher Z transform can also be viewed as a nonlinear transformation that enhances the extremely correlated or anti‐correlated values and suppresses the values in between. The range of correlation is transformed from [−1, 1] to $\left[-\infty ,\infty \right].$

#### 2.5.2 Degree normalization

Degree normalization, also known as adjacency matrix normalization, is a technique used in graph theory where the n ROIs are assumed as a node of a graph and the correlation values are assumed as the weight of the edges connecting the nodes. In this method, the correlation values of a particular node are scaled by the degree of the node, that is, sum of the correlation values between the given node and all other nodes. Let the Pearson's Correlation matrix here assumed to be Adjacency matrix be denoted as $A\in {\mathbb{R}}^{n\times n}$. Without loss of generality, the self‐loops (or self‐correlation) are ignored. To avoid complex values, the unsigned version of A is considered by taking the entry‐wise absolute value of the correlation coefficients in A denoted as A. The normalized correlation matrix $A_{\text{degree}}$ is obtained as

(2) $$A_{\text{degree}}=B^{-\frac{1}{2}}\left|A\right|B^{-\frac{1}{2},}$$

where $B\in {\mathbb{R}}^{n\times n}$ is given by,

$$B_{i,i}=b_{i}$$

$$B_{i,j}=0\forall i\neq j$$

and $b_{i}$ is the degree computed as,

$$b_{i}=\sum_{j=1}^{n}{\left|A\right|}_{i,j}.$$

Degree normalization modulates any excessive influence of the nodes by its corresponding weighted degree. It is worth noting that the range of values in the degree normalized matrix is from 0 to 1; however, due to the division by degree, the values tend to become relatively small. (Refer to Figure S1 for the flowchart of computation of the degree normalization).

### 2.6 Train‐Test Split

Of the 10 FC vectors per subject, we used five for training the DL algorithms, and the other five were used for testing. Effectively, we have a data matrix for training $Y_{i}\in {\mathbb{R}}^{\tilde{n}\times 5}$ and for testing $\tilde{Y_{i}}\in {\mathbb{R}}^{\tilde{n}\times 5}$ for the $i^{\mathrm{th}}$ subject.

Further, we ran the algorithms for every possible combination of scan in train and test matrix to check the stability and reproducibility, that is, discussed in Section 3.10.

### 2.7 Dictionary learning algorithms

DL is a technique used in machine learning and signal processing to extract a set of finite features known as atoms, that can represent the data. Based on the DL algorithm used, a linear combination of atoms can represent the data in a compact form. Using the compact or sparse representation, the common or the subject‐specific component of the data can be extracted.

In general, DL algorithms aim to express a given signal y as a linear combination of atoms. The collection of atoms is defined as a dictionary D and the signal y can be written as

(3) y=Dx,

where x is the coefficient vector depicting the weights of each atom toward the construction of signal. The available dictionaries in the literature can be classified into two categories namely: analytic and data‐driven. In the first category, the dictionary is defined using mathematical expressions, such as Fourier Transform, Cosine Transform, and so on, which does not depend on the data. The second category uses the data (example signals) to derive the atoms of the dictionary. The examples here are PCA, ICA, K‐Singular Value Decomposition (KSVD), and so on. In the literature, data‐driven dictionaries are preferred over analytic dictionaries hence we focus only on the data‐driven dictionaries. For a collection of q different signals Equation (3) can be written as

(4) Y=DX.

Here $Y=\left[y_{1},y_{2},y_{3},\ldots ,y_{q}\right]$ and $X=\left[x_{1},x_{2},x_{3}\ldots ,x_{q}\right]$. We briefly describe 4 such DL algorithms namely, PCA, RPCA, KSVD, and COBE and their application to compute the individual specific components from the given network specific FC.

#### 2.7.1 Principal component analysis (PCA)

PCA is a widely used method for dimensionality reduction in the area of pattern recognition. It can be considered as one of the DL approaches, where the dictionary is formed using eigenvectors (also called principal components [PCs]) of the data's covariance matrix. Which makes the data uncorrelated in the transformed space. Here, a given observation can be written as a linear combination of PCs. Amico and Goñi (2018) showed how to maximize the individual fingerprint in the FC domain by reconstructing the FCs by an optimal number of PCs.

Here we concatenate every subject's data matrix to one global data matrix as follows,

(5) $$Y=\left[Y_{1},Y_{2},Y_{3},\ldots ,Y_{p}\right]\in {\mathbb{R}}^{\tilde{n}\times 5p}.$$

Here, p is the total number of subjects. First, the mean $\bar{y}$ is subtracted from each row $Y^{\prime }=Y-\bar{y}$ and then the eigen vectors and the eigen values are estimated from the covariance matrix of $Y^{\prime }$. The mean subtracted matrix $Y^{\prime }$ can be decomposed as follows,

(6) $$Y^{\prime }=D_{\mathrm{pca}}X_{\mathrm{pca}}$$

where $D_{\mathrm{pca}}=\left[e_{1},e_{2},e_{3},\ldots ,e_{\tilde{n}}\right]$ and $e_{i}$ are the eigen vectors. $X_{\mathrm{pca}}$ is the corresponding coefficient matrix estimated by $X_{\mathrm{pca}}=D_{\mathrm{pca}}^{T}Y^{\prime }$. Moreover, the subject‐specific component ($\mathrm{Su}b_{\mathrm{pca}}$) can be estimated by choosing $\tilde{m}\;\left(\tilde{m}<\tilde{n}\right)$ eigen vectors corresponding to the $\tilde{m}$ highest eigen values as follows,

(7) $$\mathrm{Su}b_{\mathrm{pca}}=D_{\mathrm{pca}}^{\tilde{m}}X_{\mathrm{pca}}^{\tilde{m}}.$$

Here $D_{\mathrm{pca}}^{\tilde{m}}=\left[e_{1},e_{2},e_{3},\ldots ,e_{\tilde{m}}\right]$ and $X_{\mathrm{pca}}^{\tilde{m}}$ is the corresponding coefficient matrix.

**FIGURE 2:**
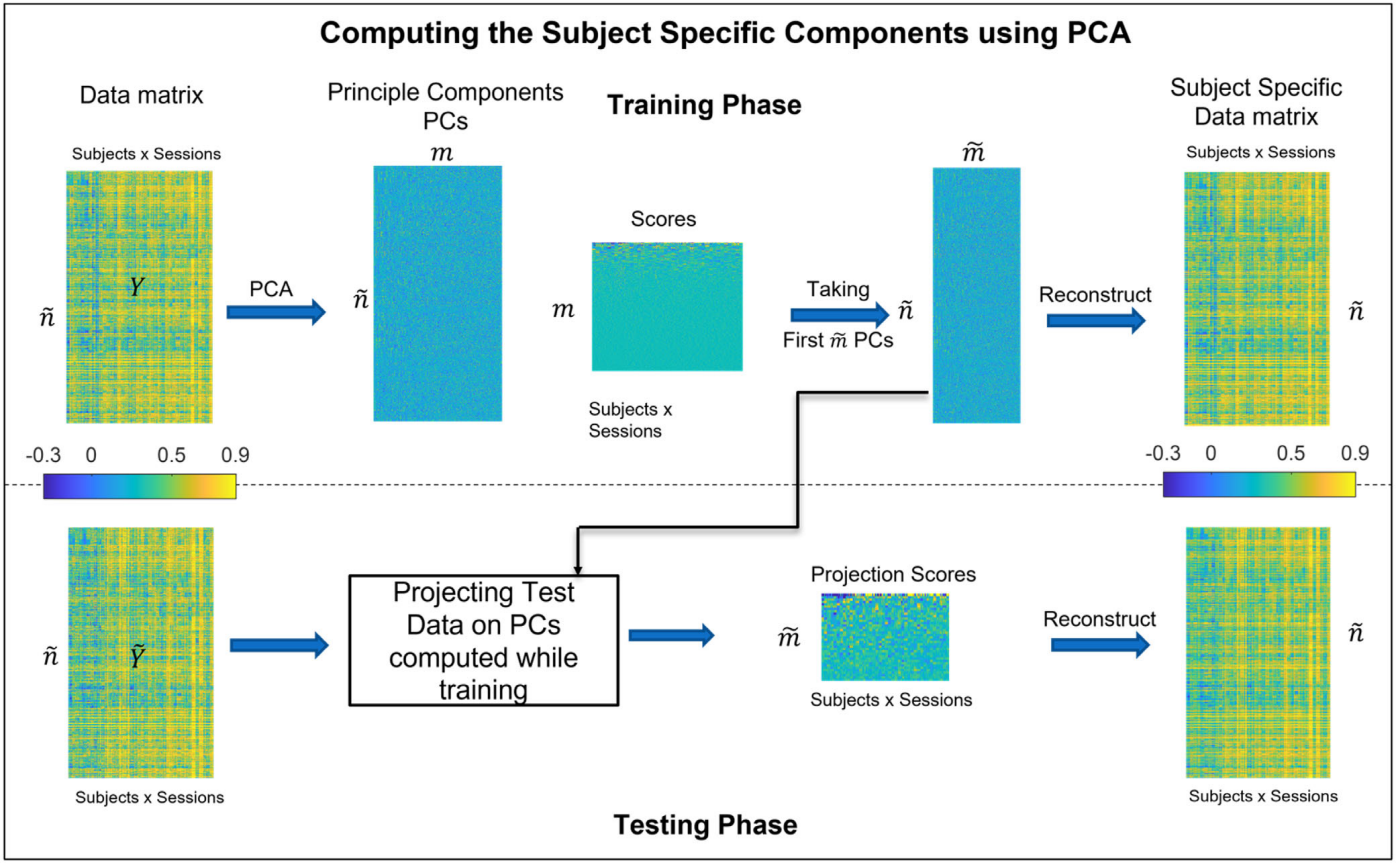
The Training data matrix Y is given as input to the PCA algorithm, which gives **m** principal components (PCs). First $\tilde{m}$ PCs are chosen, and the data are reconstructed to get the subject‐specific components. During testing, the test data $\tilde{Y}$ are projected over the PCs computed while training, and the reconstructed matrix is the subject‐specific data matrix.

During the testing phase, the testing data matrix ${\tilde{Y}}_{i}$ is first concatenated to make a global test matrix ${\tilde{Y}}_{i}$ which is then subtracted by the mean vector $\bar{y}$ found at the training phase and further projected on the eigenvectors $D_{\mathrm{pca}}^{\tilde{m}}$. Furthermore, the projected data are reconstructed back in the original space, which gives us the subject‐specific components. Figure 2 summarizes the training and the testing phase of PCA to find the individual differences.

#### 2.7.2 Robust PCA (RPCA)

It has been observed that PCA is sensitive to noise and corrupted observations, as PCA minimizes the squared error between the original data and the PCA reconstructed data. Candes et al. (2009) addresses this issue by a method Robust PCA (RPCA), which attempts to minimize the absolute value of the error, which makes RPCA robust to noise.

This approach decomposes the global FC data matrix Y as

(8) $$Y=L_{\text{rpca}}+S_{\text{rpca}},$$

where $L_{\text{rpca}}$ is a low‐rank matrix and $S_{\text{rpca}}$ is sparse, both have the same dimensions as that of Y. Further, as described by Qin et al. (2019), an Online Dictionary learning (ODL) (Mairal et al., 2010) algorithm is trained on the sparse connectivity traits matrix $S_{\text{rpca}}$ after making it zero mean and unit variance, giving us the basis that can determine the traits matrix for any test scan. ODL attempts to decompose the traits matrix as follows,

(9) $$S_{\text{rpca}}=D_{\text{rpca}}\alpha_{\text{rpca}}.$$

Here $D_{\text{rpca}}\in {\mathbb{R}}^{\tilde{n}\times k}$ is the basis Dictionary, k is the number of basis, and $\alpha_{\text{rpca}}$ is the corresponding coefficient matrix. The $D_{\text{rpca}}$ matrix is stored, which can generate the trait matrix for any test scan. In this study, we have used the implementation provided by Aravkin et al. (2014) for the RPCA algorithm and the SPAMS toolbox for ODL computation (Mairal et al., 2010). Qin et al. (2019) also attempted to find the subject‐specific components in a similar manner.

In the testing phase, least squares are used to get the coefficients ${\tilde{\alpha }}_{\text{rpca}}$ from the stored $D_{\text{rpca}}$ basis for the global test matrix $\tilde{Y}$ as described in Qin et al. (2019).

(10) $${\mathrm{Sub}}_{\text{rpca}}=D_{\text{rpca}}\;{\tilde{\alpha }}_{\text{rpca}}$$

**FIGURE 3:**
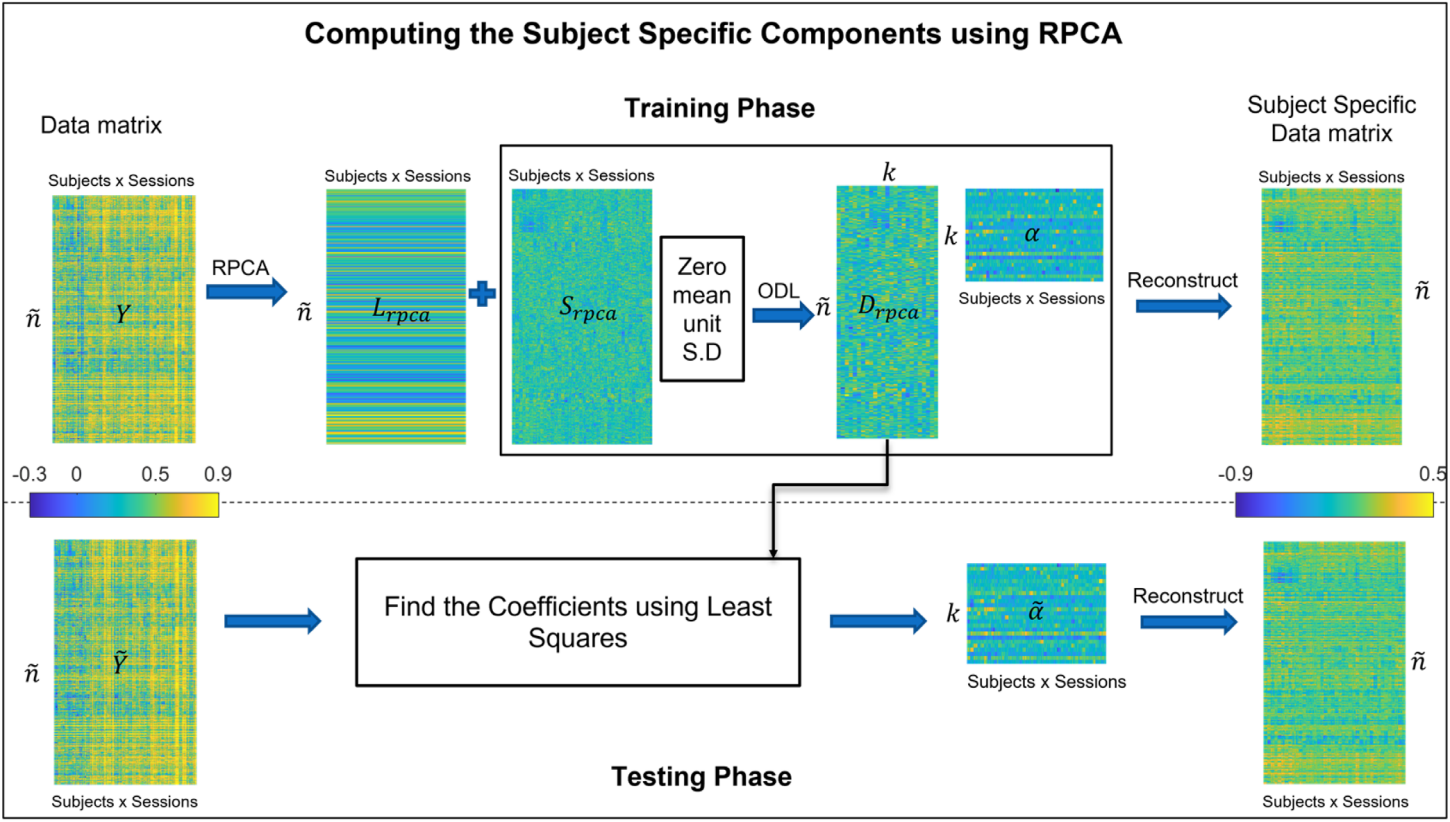
RPCA training and testing overview. The data matrix Y goes through the RPCA algorithm during the training phase, which decomposes Y into a low‐rank matrix $L_{\text{rpca}}$ and a sparse matrix $S_{\text{rpca}}$. $S_{\text{rpca}}$ having the individual differences is further decomposed by Online Dictionary Learning (ODL) which finds out the basis $D_{\text{rpca}}$ whose linear combination can construct the individual components to any data matrix. This gives the subject‐specific components for training. During the test, least squares are used to get the coefficients ${\tilde{\alpha }}_{\text{rpca}}$ for the test matrix $\tilde{Y}$ which on reconstruction gives the subject‐specific component.

The overview of the training and testing phase of the RPCA algorithm is summarized in Figure 3.

#### 2.7.3 K‐SVD

K‐SVD is a well‐known DL algorithm for obtaining sparse codes over a dictionary to reconstruct the data matrix. It is an iterative method that alternates between sparse coding of the examples based on the current dictionary and then updating the dictionaries using Singular value decomposition (SVD) to fit the data (Aharon et al., 2006). Recently, Cai et al. (2019) demonstrated that subtracting the data matrix Y from its approximate matrix using KSVD, brings out the individual components in the FC vectors.

K‐SVD is performed on the global data matrix formed by concatenating all the subject's data matrix (refer Equation 5). K‐SVD decomposes the global data matrix Y as follows,

(11) $$Y=D_{\text{ksvd}}X_{\text{ksvd}}$$

where $D_{\text{ksvd}}\in {\mathbb{R}}^{\tilde{n}\times k}$ denotes the dictionary consisting of k dictionary atoms and $X_{\text{ksvd}}\in {\mathbb{R}}^{k\times \left(5\times p\right)}$ denotes the corresponding sparse codes learnt by the K‐SVD algorithm. The dictionaries are initialized randomly, and the sparse codes with sparsity $s_{0}$ are learned by Orthogonal Matching Pursuit (OMP). Further, the *k*‐dictionary atoms are updated one by one using SVD. Least squares are used to get the coefficient matrix ${\tilde{X}}_{\text{ksvd}}$ for the global testing data matrix $\tilde{Y}$ using the dictionaries $D_{\text{ksvd}}$. We used the implementation provided by Aharon and Elad (2008) for OMP. The individual components for the test are computed by subtracting the reconstructed data matrix from the test data matrix as follows,

(12) $$\mathrm{Su}b_{\text{ksvd}}=\tilde{Y}-D_{\text{ksvd}}X_{\text{ksvd}}.$$

**FIGURE 4:**
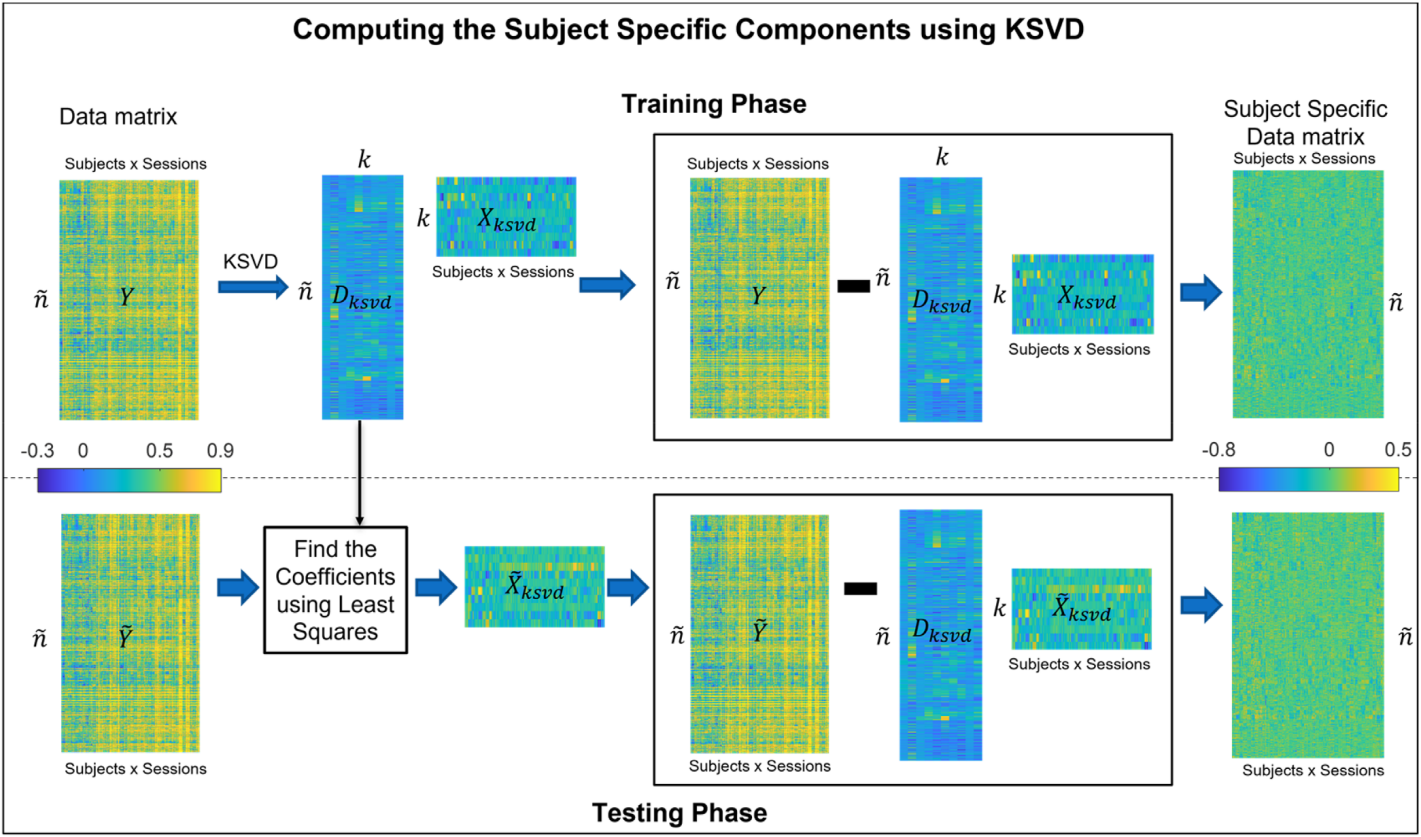
K‐SVD training and testing overview. K‐SVD algorithm decomposes the data matrix Y into a dictionary $D_{\text{ksvd}}$ and sparse coefficients X. The product $D_{\text{ksvd}}X_{\text{ksvd}}$ represents the common or shared information. This, when subtracted with the data matrix Y gives the subject‐specific component. During testing, least squares are used to find the coefficients for the Dictionary obtained during the test. This, when subtracted from the Test data matrix $\tilde{Y}$, gives us the Subject‐specific matrix for the test.

Figure 4 shows the Block diagram summarizing the training and testing phase of K‐SVD.

#### 2.7.4 Common orthogonal basis extraction

COBE was first described by Zhou et al. (2016). It works on multiblock data where data are a collection of matrices. COBE tries to find an orthogonal basis that is shared by all the matrices in the data. Kashyap et al. (2019) demonstrated how the algorithm could be applied to rs‐fMRI data. They have demonstrated the efficacy of the COBE algorithm on the averaged BOLD signal using a combination of Schaefer 400 nodes Atlas and 19 subcortical regions (Fischl et al., 2002), while in this study, we use it on the FC vectors. In this approach, individual matrix is decomposed as,

(13) $$Y_{i}=D_{\text{cobe}}X_{\text{cobe}}^{i}+D_{\text{cobe}}^{i}{{\bar{X}}^{i}}_{\text{cobe}}.$$

Here, $D_{\text{cobe}}\in {\mathbb{R}}^{\tilde{n}\times C}$ is the shared dictionary containing C atoms common to all subjects and $D_{\text{cobe}}^{i}\in {\mathbb{R}}^{\tilde{n}\times C_{i}}$ is the subject‐specific dictionary containing $C_{i}$ atoms for the $i^{\mathrm{th}}$ subject. $X_{\text{cobe}}^{i}\in {\mathbb{R}}^{C\times 5}$ and ${{\bar{X}}^{i}}_{\text{cobe}}\in {\mathbb{R}}^{C_{i}\times 5}$ are the corresponding coefficients of the shared and subject‐specific dictionaries. However, the COBE algorithm focuses on getting the shared dictionary $D_{\text{cobe}}$ and its coefficients $X_{\text{cobe}}^{i}$. The subject‐specific component $D_{\text{cobe}}^{i}{{\bar{X}}^{i}}_{\text{cobe}}$ is later found by subtracting the shared component from the data matrix $Y_{i}$ as follows,

(14) $$D_{\text{cobe}}^{i}{{\bar{X}}^{i}}_{\text{cobe}}=Y_{i}-D_{\text{cobe}}X_{\text{cobe}}^{i}.$$

The shared dictionary $D_{\text{cobe}}$ is stored in memory to get the individual differences in the test scans.

An important point that distinguishes the COBE algorithm from the algorithms discussed before is that here the subject data matrix $Y_{i}\in {\mathbb{R}}^{\tilde{n}\times 5}$ containing the five sessions of the subject is given and not the global matrix Y, which makes COBE a supervised algorithm.

In the testing phase, least squares are used to acquire the coefficients ${\tilde{X}}_{\text{cobe}}^{i}$ corresponding to the shared dictionary $D_{\text{cobe}}$ for the global test data matrix $\tilde{Y}$. After which we subtract the reconstructed shared information from the original data matrix to obtain the subject‐specific information as follows,

(15) $$\mathrm{Su}b_{\text{cobe}}=\tilde{Y}-D_{\text{cobe}}{\tilde{X}}_{\text{cobe}}.$$

**FIGURE 5:**
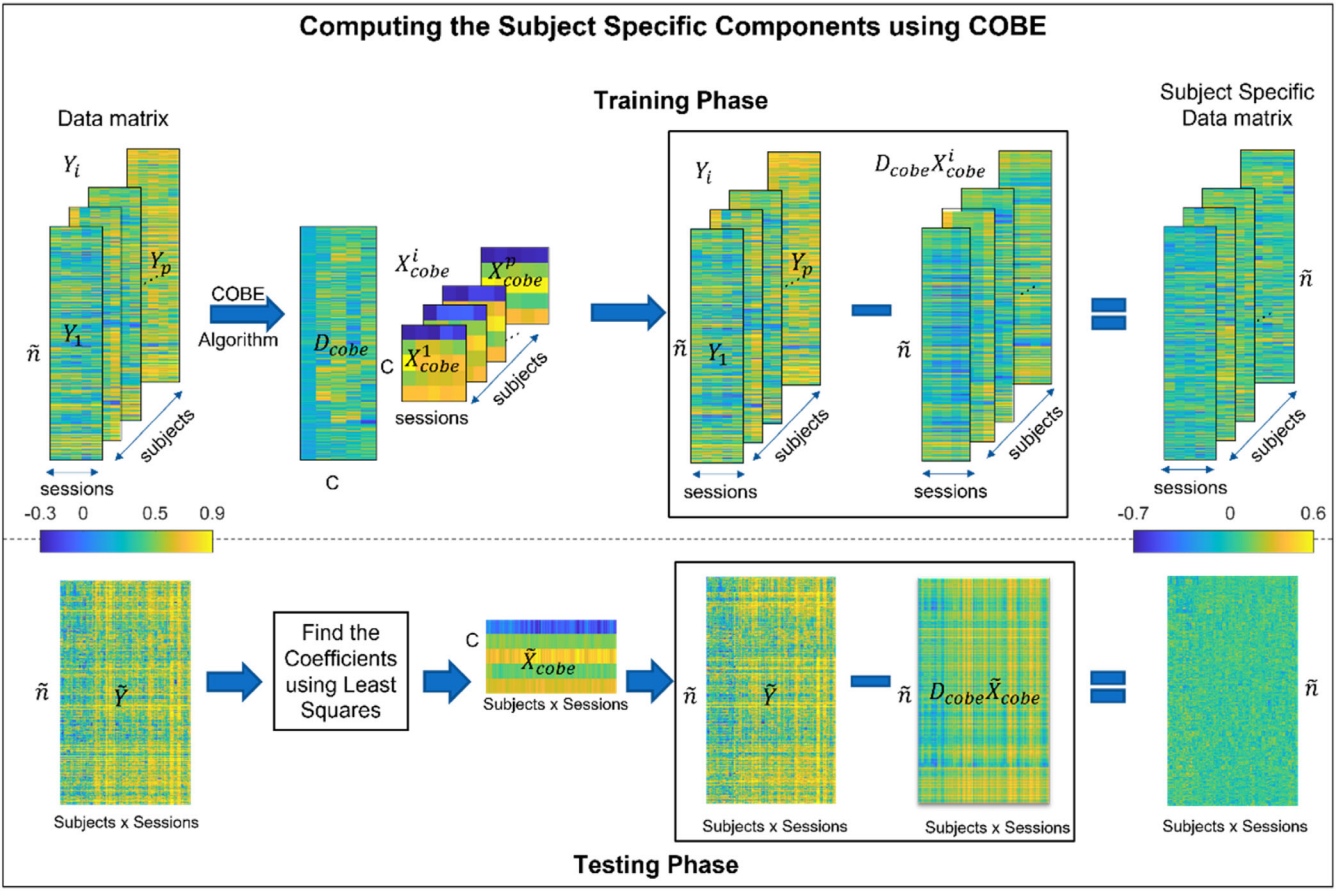
COBE training and testing overview. Here instead of the Global data matrix Y the subject data matrix $Y_{i}$ is given individually to the COBE algorithm which decomposes the matrix into a dictionary $D_{\text{cobe}}$ and coefficients matrix $X_{\text{cobe}}^{i}$ for every subject. The product $D_{\text{cobe}}X_{\text{cobe}}^{i}$ is subtracted from $Y_{i}$ for every subject which gives the subject‐specific components. During test, the least‐squares algorithm is used to get the coefficients for the dictionary $D_{\text{cobe}}$ obtained during training. Finally, the subject‐wise subtraction of the reconstructed matrix with the test data matrix ${\tilde{Y}}_{i}$ gives the subject‐specific matrix during test.

Figure 5 shows the flowchart describing the training and testing phase of the COBE algorithm to find the subject‐specific component.

### 2.8 Metrics for evaluating the subject‐specific components

This section discusses the metrics used to evaluate the subject‐specific components computed by the DL algorithms.

#### 2.8.1 Differential identifiability quality function $\left(I_{\text{diff}}\right)$

**FIGURE 6:**
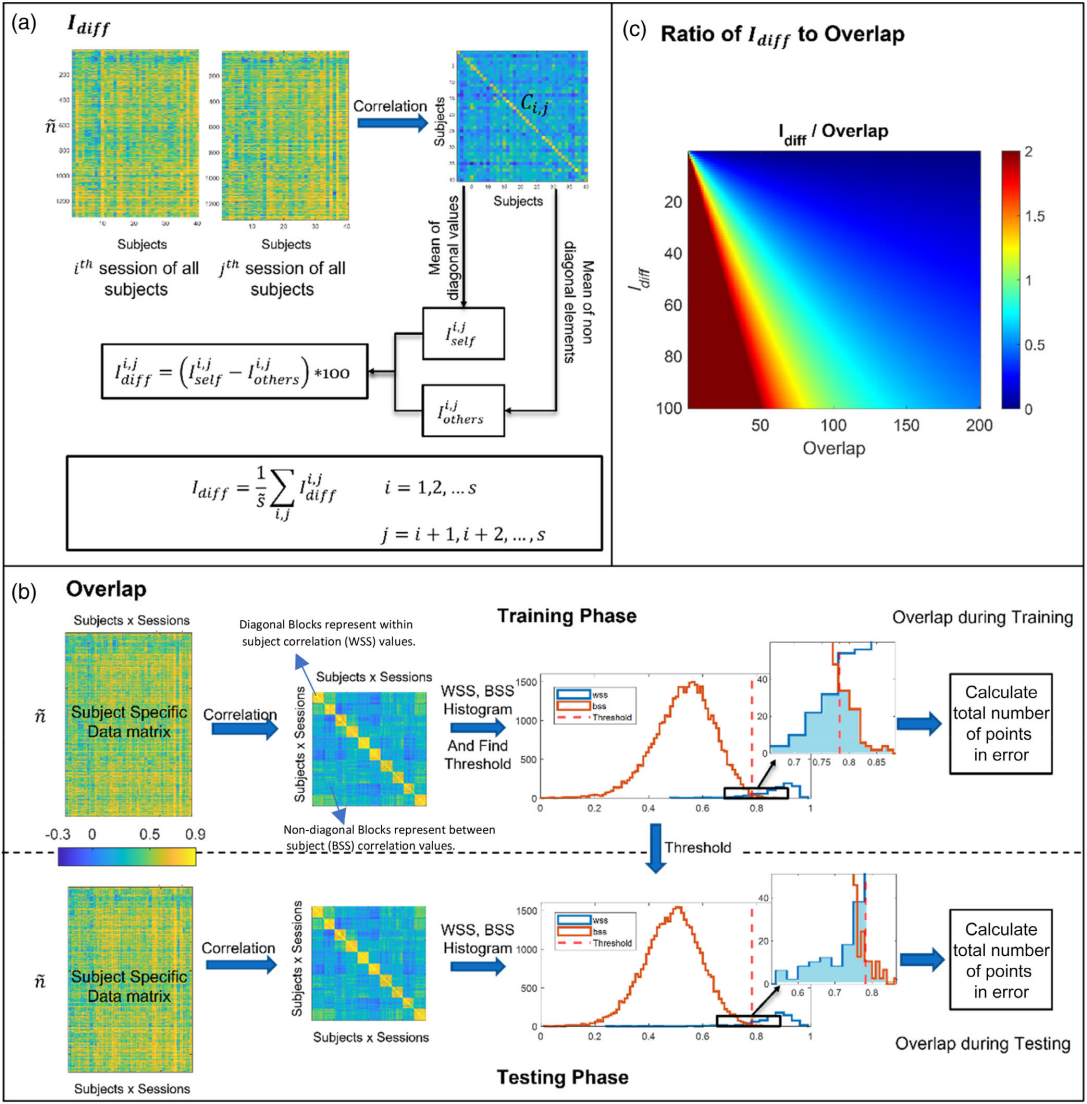
(a) $I_{\text{diff}}$ computation: The subject‐specific data matrix consisting of $i^{\mathrm{th}}$ and the $j^{\mathrm{th}}$ sessions of all the subjects. Further $C_{i,j}$ is computed by taking Pearson correlation. $I_{\text{self}}$ is the mean of diagonal elements and $I_{\text{others}}$ is the mean of non‐diagonal elements of the correlation matrix obtained in b. $I_{\text{diff}}^{i,j}$ is computed using Equation (16). Finally, $I_{\text{diff}}$ is computed as mean of the $I_{\text{diff}}$ values computed taking every ${\left(i,j\right)}^{\mathrm{th}}$ pair. (b) Overlap computation: A Pearson correlation matrix is computed from the subject‐specific data matrix. This correlation matrix was partitioned into $p^{2}$ sub‐matrices each of size s×s, then the histogram of the diagonal blocks would represent within‐subject similarity (WSS) values and the histogram of non‐diagonal blocks would represent between‐subject similarity (BSS) values. Using these histograms, a threshold is computed such that any value above the threshold is considered within‐subject and below the threshold is considered between‐subject. The threshold that minimizes the error is taken and stored in memory during the training phase. During the testing phase, the threshold computed during training is used. The total number of values in error is called Overlap. (c) Ratio of $I_{\text{diff}}$ to Overlap: Plot shows the variation of the ratio to with the variation of $I_{\text{diff}}$ and Overlap.

This metric is taken from Amico and Goñi (2018), it is calculated as follows. Considering p number of subjects and s number of sessions per subject, the sessions of every subject are arranged together such that we get s different matrices each consisting of the $i^{\mathrm{th}}$ session (i=1,2,3,…,s) of every subject. The size of these matrices is $\tilde{n}\times p$. Considering two matrix at a time we will have $\tilde{s\;}=s\times \frac{s-1}{2}$ different combinations. For each combination, let $C_{i,j}\;i=1,2,\ldots ,s$, j=i+1,i+2,…,s be the “identifiability matrix,” which is computed by talking a correlation of the two matrices containing $i^{\mathrm{th}}$ and the $j^{\mathrm{th}}$ sessions of every subject. The dimension of $C_{i,j}$ is p×p (see Figure 6a), where p is the number of subjects in the database. Let $I_{\text{self}}^{i,j}$ represents the average of the main diagonal elements of $C_{i,j}$, which consist of the Pearson correlation values between visits of the same subjects. Similarly, let $I_{\text{others}}^{i,j}$ defines the average of the off‐diagonal elements of matrix $C_{i,j},$ that is, the correlation between visits of different subjects. Then the differential identifiability ($I_{\text{diff}}^{i,j}$) is defined as the difference between both terms as,

(16) $$I_{\text{diff}}^{i,j}=\left(I_{\text{self}}^{i,j}-I_{\text{others}}^{i,j}\right)\times 100.$$

We get $\tilde{s}$ such $I_{\text{diff}}^{i,j}$ scores. The mean of these scores over all $\tilde{s}$ pairs is the $I_{\text{diff}}$ score.

$$I_{\text{diff}}=\frac{1}{\tilde{s}}\sum_{i,j}I_{\text{diff}}^{i,j}$$

It should be noted that a greater $I_{\text{diff}}$ score, signifies better estimate of individual components. Ideally $I_{\text{diff}}$ should be 100.

#### 2.8.2 Overlap

Assuming p subjects and s sessions per subject, there are p×s individual FC vectors in all. The hypothesis is that the individual‐specific FC vectors should be similar within the same subject and different across different subjects. The Pearson correlation of the estimated individual‐specific FC vectors between every scan of every subject can be computed and obtain a $\left(\mathrm{ps}\right)\times \left(\mathrm{ps}\right)$ matrix. If this correlation matrix is partitioned into $p^{2}$, s×s matrices, the diagonal blocks represent the within‐subject correlation values, and the other blocks represent the between‐subject correlation values (see Figure 6b). From this, a histogram of the between subject's correlation value and within subject's correlation value can be computed. Using the histogram, a threshold can be found, which separates the within and between‐subject distributions. This threshold can help identify whether a test scan belongs to the same subject or not. After thresholding, the total number of correlations that are misclassified or, in other words, the overlap between the within‐subject and the between‐subject distributions is used to determine how good the subject‐specific component is. The less the Overlap, the better the individuality in the subject‐specific component. The optimal threshold that minimizes the Overlap is saved for each DL algorithm during the training phase. This threshold is then used during the testing phase to determine the overlap during the test. A less value of Overlap is desirable. Ideally Overlap should be 0.

#### 2.8.3 Ratio of $I_{\text{diff}}$ to Overlap

$I_{\text{diff}}$ looks at the mean of within and between subjects' correlation value, hence for $I_{\text{diff}}$ to be high, the means of within‐subject correlation and the between‐subject correlation should be far apart. Since $I_{\text{diff}}$ only accounts for the mean and not the variance, it is possible to have a reasonably good $I_{\text{diff}}$, but a high value of Overlap. For example, consider $I_{\text{others}}$ = 0.3 and the between‐subject distribution to have a large variance, due to this there is a good chance that some of the between‐subject correlations are close to 1. Similarly, say $I_{\text{self}}$ = 0.7 but with a large variance of within‐subject correlation there can be quite a few values that are close to 0; Although, in this case, the means of both the distribution are far, resulting in a good $I_{\text{diff}}$ (40 in this case), Overlap can be high and thus it is a better metric as it shows the actual error one can get while identifying within or between subject correlations. On the other hand, $I_{\text{diff}}$ explains the separation between the means of within‐subject and between‐subject distribution while overlap does not. We not only want to minimize the overlap of the two distributions, but also want the distributions to be far apart, as, the more they are apart in training, it is probable that they will be apart during testing. So, if for two cases the overlap is the same, we want to give a higher score to the case having the means of the two distributions farther which can be done using $I_{\text{diff}}$. Both cases are essential. The former says there should be one threshold that can differentiate the within and between subjects with minimum error, while the latter says the means of the within‐subject, and the between‐subject correlations should be far apart for better repeatability. To account for both, we take the ratio of $I_{\text{diff}}$ to Overlap, which must be maximized. Both the extent of separation of the means of $I_{\text{self}}$ and $I_{\text{others}}$ and the classification error of within and between‐subject correlations are taken into consideration here. The ratio will have a higher value if there are less misclassifications (lower Overlap) and the separation between the means of within and between subjects' distribution is large (higher $I_{\text{diff}}$). See Figure 6c which shows the variation of the ratio as $I_{\text{diff}}$ and Overlap are varied.

## 3 RESULTS

One subject from the HNU dataset and one from the MSC dataset was excluded as they had motion displacements greater than 3 mm. For this study, we used the Schaefer atlas to determine 400 ROIs. For each ROI, the number of voxels time courses was averaged to find a representative time course corresponding to the ROI. This was done for all the 400 ROIs.

### 3.1 $I_{\text{diff}}$, Overlap, ratio of $I_{\text{diff}}$ to Overlap before applying any algorithm

The $I_{\text{diff}}$ and Overlap for the Fisher Z transformed raw correlation values of the fronto‐parietal network (FPN) and Somato Motor network (SMN) were 32 and 26, 262, and 432, respectively (refer to Figure S2). The greater $I_{\text{diff}}$ for FPN suggests that the difference between the mean value of self‐individuality ($I_{\text{self}}$) (similar to mean within‐subject correlation) and $I_{\text{other}}$ (similar to mean between‐subject correlations) for FPN is greater than SMN. The Overlap values suggest that FPN is better able to classify the within and between‐subject correlations with fewer errors as compared to the SMN. Ideally, a high value of $I_{\text{diff}}$ signifies that the means of $I_{\text{self}}$ and $I_{\text{other}}$ are as far as possible and on the other hand, an Overlap of 0, would mean that no within or between subject correlations are misclassified.

For FPN and SMN, the ratio of $I_{\text{diff}}$ to Overlap with Fisher Z normalization was 0.12 and 0.06, respectively. This suggests that FPN is better as compared to SMN in finding the subject‐specific information.

Based on the above‐mentioned analysis we found that the FPN for degree normalization had the highest value of the ratio of $I_{\text{diff}}$ to Overlap (0.2) among the 7 Yeo networks followed by default mode network (DMN) with a value of 0.16 and dorsal attention network (DAN) with a value of 0.35. Limbic network (LN), visual network (VN), SMN, and ventral attention network (VAN) had relatively lower values of the ratio.

### 3.2 Parameter search

Here we discuss how the parameters for the different dictionary algorithms effect the ratio of $I_{\text{diff}}$ to Overlap.

#### 3.2.1 PCA

**FIGURE 7:**
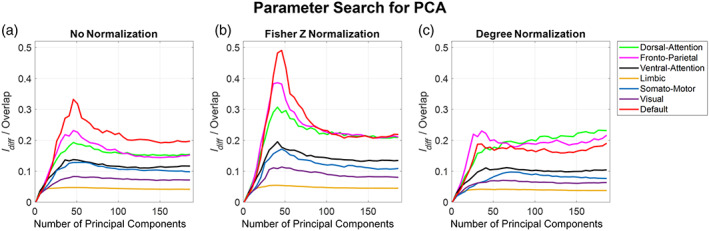
Number of Principle components (PC) used versus the ratio of $I_{\text{diff}}$ to Overlap. Plots show how the ratio changes as we change the number of PCs used to reconstruct the train data matrix Y. The number of PCs varied from 1 to 189 in steps of 5. (a–c) The effect of the different normalization methods on the ratio.

PCA has only one parameter that we can tune to maximize the ratio of $I_{\text{diff}}$ to Overlap which is the number of PCs used to reconstruct the train data matrix Y. Figure 7 shows the variation of the ratio of $I_{\text{diff}}$ to Overlap with respect to the change in number of PC used for reconstruction. The number of PCs was varied from 1 to 189 for every RSN and with different normalization methods. Referring to Figure 7, the ratio initially increased as the number of PC were increased, it reached a peak around 50 components and then decreased as the number of PC were increased. DMN was observed to be a better network as it gave the highest value for the ratio due to its low value for overlap (around 100 and $I_{\text{diff}}$ around 35 with no normalization and Fisher Z), but FPN was observed to be better if degree normalization is considered as it achieved a higher $I_{\text{diff}}$ value of 40. The LN, however, achieved the lowest value of the ratio as compared to any other Yeo rs‐networks as it had the maximum Overlap value (around 700) across all normalization methods and a lowest $I_{\text{diff}}$ (about 28) with no normalization and Fisher Z normalization and around 25 with degree normalization.

We also looked at the percentage of variance explained by each PC. We observed the first 50 PCs explained about 93% of variance for the LN when no normalization or Fisher Z normalization was used but with degree normalization, only 86% of variance was explained. However, with the DMN the first 50 components explained 86% variance using no normalization or Fisher Z normalization, and with degree normalization that fell to 79%. FPN also gave results similar to the DMN. The other networks had a lesser percentage of variance explained by the first 50 components compared with the LN but greater than the DMN (refer to Figure S3).

#### 3.2.2 R‐PCA

**FIGURE 8:**
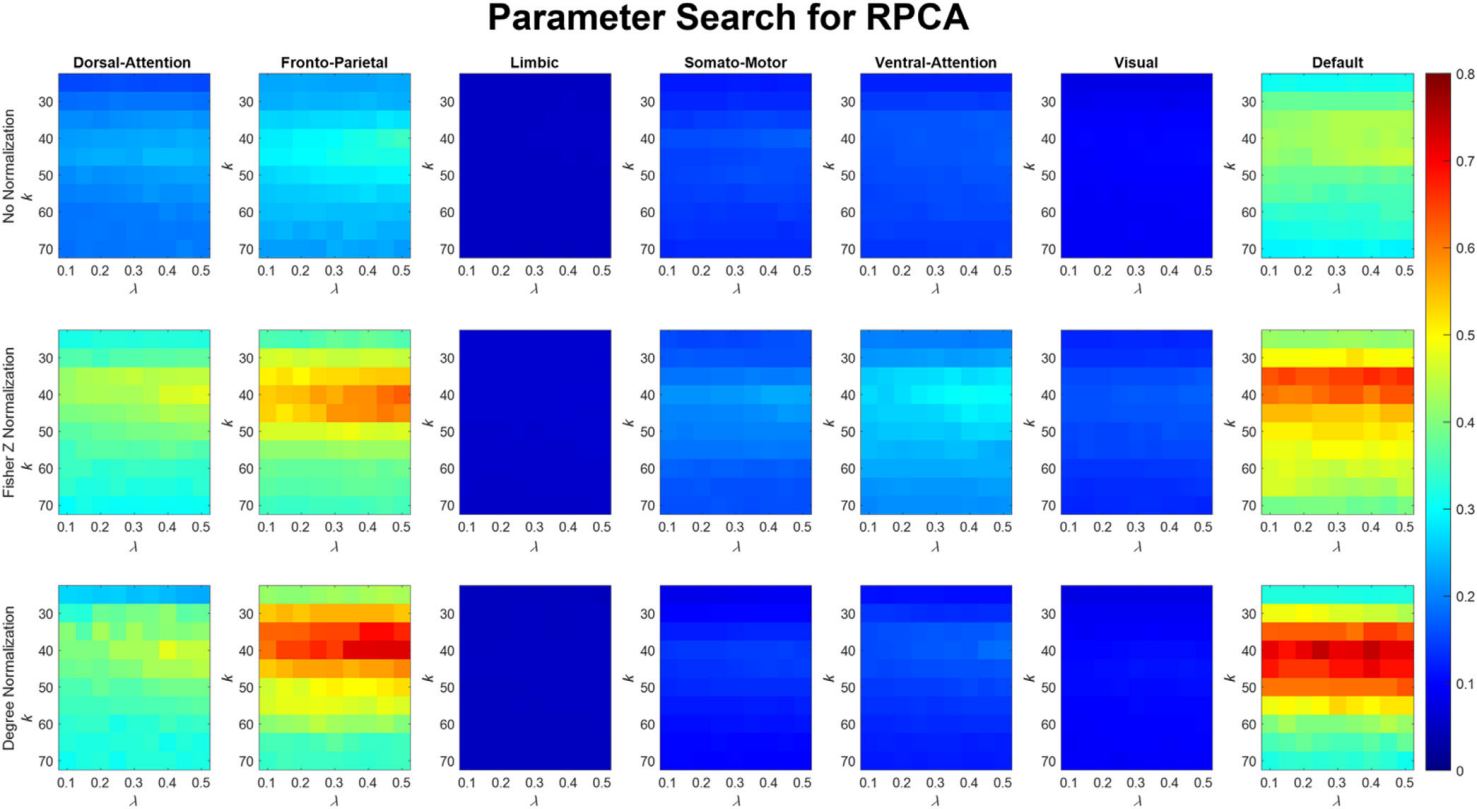
Effect of changing the number of atoms (k) and the λ parameter of the dictionary computed by the Online dictionary learning algorithm on the Sparse connectivity Traits matrix obtained by the R‐PCA algorithm on the training data matrix Y. The number of dictionary atoms k is varied from 25 to 70 in steps of 5, and the λ parameter is varied from 0.1 to 0.5 in steps of 0.05.

Although R‐PCA does not have any parameters that can be tweaked, the ODL algorithm applied on the sparse connectivity matrix obtained from R‐PCA had two parameters that are, (1) k: number of atoms in the dictionary and (2) λ parameter. The number of dictionary atoms k were varied from 25 to 70 in steps of 5, and we observed from Figure 8 that k in between 35 and 50, irrespective of λ, acquired a relatively high value of the ratio of $I_{\text{diff}}$ to Overlap. Especially when λ is in between 0.3 and 0.5, the ratio is at its peak. The DMN and the FPN achieve a value around 0.7 when 40 dictionary atoms are used with degree normalization. The value of the ratio for DAN is around 0.5 using 40 dictionaries. For the other networks, the value of the ratio is less than 0.2 when using degree normalization. However, with Fisher Z transform all networks, except for VAN and the SMN, do not perform as well as they perform with degree normalization.

#### 3.2.3 K‐SVD

**FIGURE 9:**
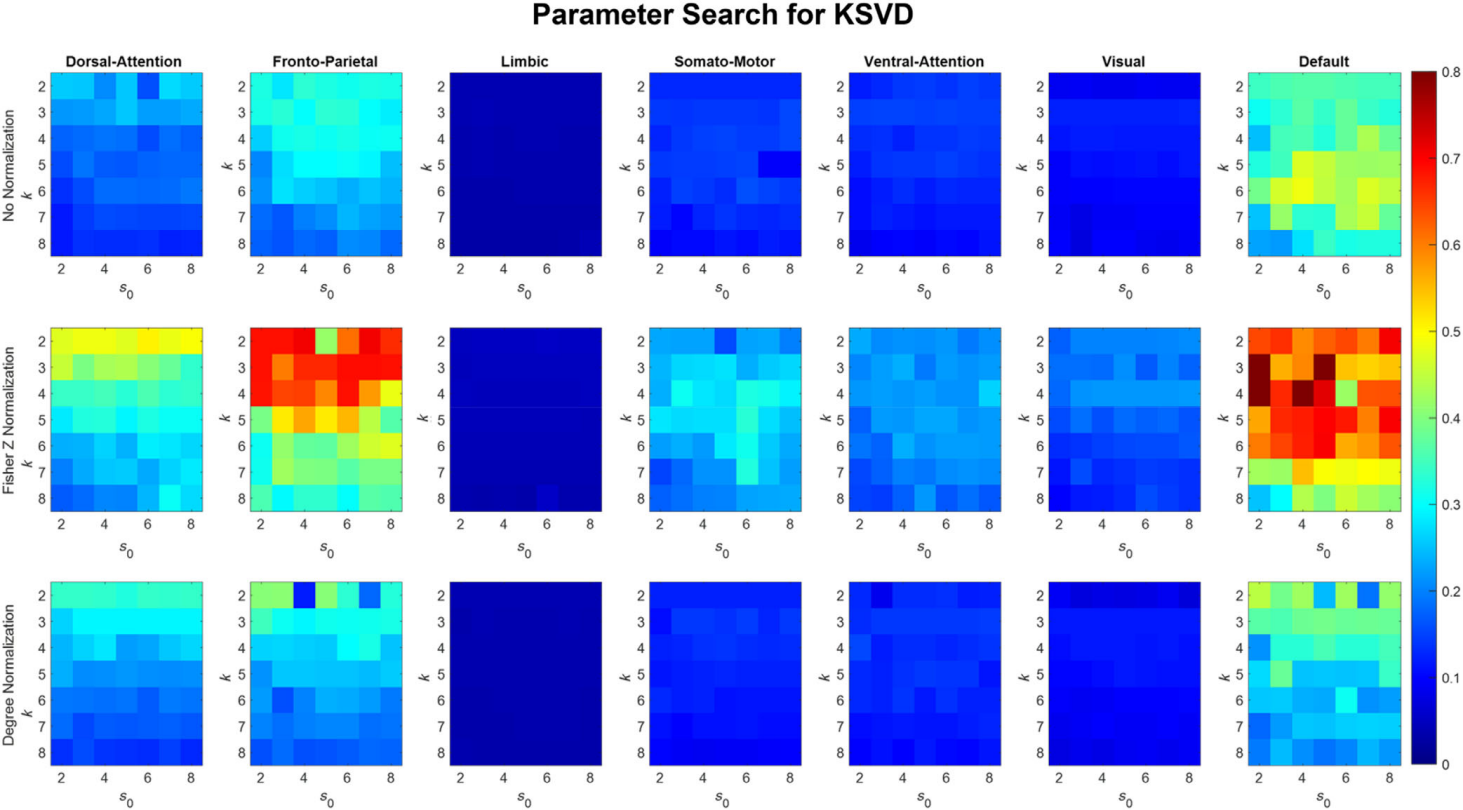
Variation of number of atoms (k) in the K‐SVD dictionary and the sparsity ($s_{0}$) in the corresponding coefficient matrix. k and $s_{0}$ is varied from 2 to 8, across all the rs‐networks and normalization methods.

The number of atoms in the K‐SVD dictionary k and the corresponding Sparsity parameter $s_{0}$ are the two parameters that had to be tuned to maximize the ratio of $I_{\text{diff}}$ to Overlap. Both k and $s_{0}$ were varied from 2 to 8 as shown in Figure 9. K‐SVD enhances the ratio to about 0.7 in FPN with $I_{\text{diff}}$ value around 44 and Overlap of 64 using two dictionary atoms and about 0.8 for the DMN ($I_{\text{diff}}$ = 42, Overlap = 52) using 3 or 4 dictionary atoms with the Fisher Z normalization. DAN is at the third place with the ratio being 0.5 ($I_{\text{diff}}$ = 44 and Overlap = 86) when two dictionary atoms were used with Fisher Z transform. LN was observed to have a value of ratio less than 0.1, because of a very high Overlap which was greater than 500 across all Normalization methods as well as all values of k and $s_{0}$, and a low $I_{\text{diff}}$ as well. Using no normalization DMN achieved a value of ratio around 0.5 using 6 dictionary atoms. FPN with no normalization achieved a value around 0.35 ($I_{\text{diff}}$ = 41 and Overlap = 116) with 3 or 4 dictionary atoms and DAN achieved a ratio of 0.25 ($I_{\text{diff}}$ = 40 and Overlap = 168) using 2 dictionary atoms. Otherwise, the values of the ratio for networks other than DMN, FPN, and DAN were mostly less than 0.2 because of a high overlap that was greater than 200 when no normalization was used. Degree normalization was observed to have higher values of the ratio in FPN, and DAN networks as compared with no normalization with DAN achieving a value around 0.3 ($I_{\text{diff}}$ = 41 and Overlap = 138) and FPN achieving a value around 0.45 ($I_{\text{diff}}$ = 43 and Overlap = 98) using two dictionary atoms.

#### 3.2.4 COBE

**FIGURE 10:**
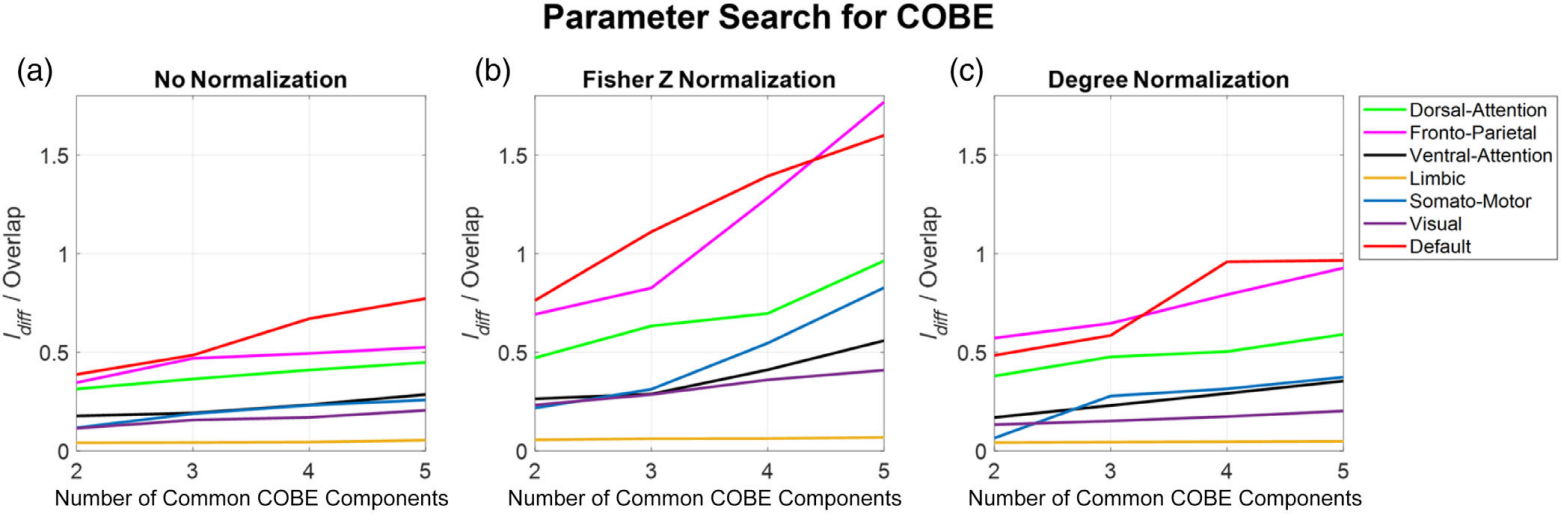
Variation of number of common components C in COBE algorithm. We can observe the change in the ratio of $I_{\text{diff}}$ to Overlap with respect to the number of common components chosen in the COBE algorithm. The number of common components was varied from 2 to 5 for all networks across all normalization methods (a–c).

COBE had only one parameter, that is the number of common components C, which could be tuned to maximize the ratio of $I_{\text{diff}}$ to Overlap (refer Figure 10). We varied the number of common components from 2 to 5 and checked the value of the ratio. Results show an increasing trend as the number of common components C was increased for all networks except the LN, which is almost constant. However, for the DMN, FPN, and the DAN networks, C increased more rapidly compared to others. DMN and FPN achieved the highest value of the ratio which is a little greater than 1.5 with the Fisher Z transformation. Taking C as 5 gave the best results across all networks as well as normalization methods.

### 3.3 Comparison between the DL algorithms

**FIGURE 11:**
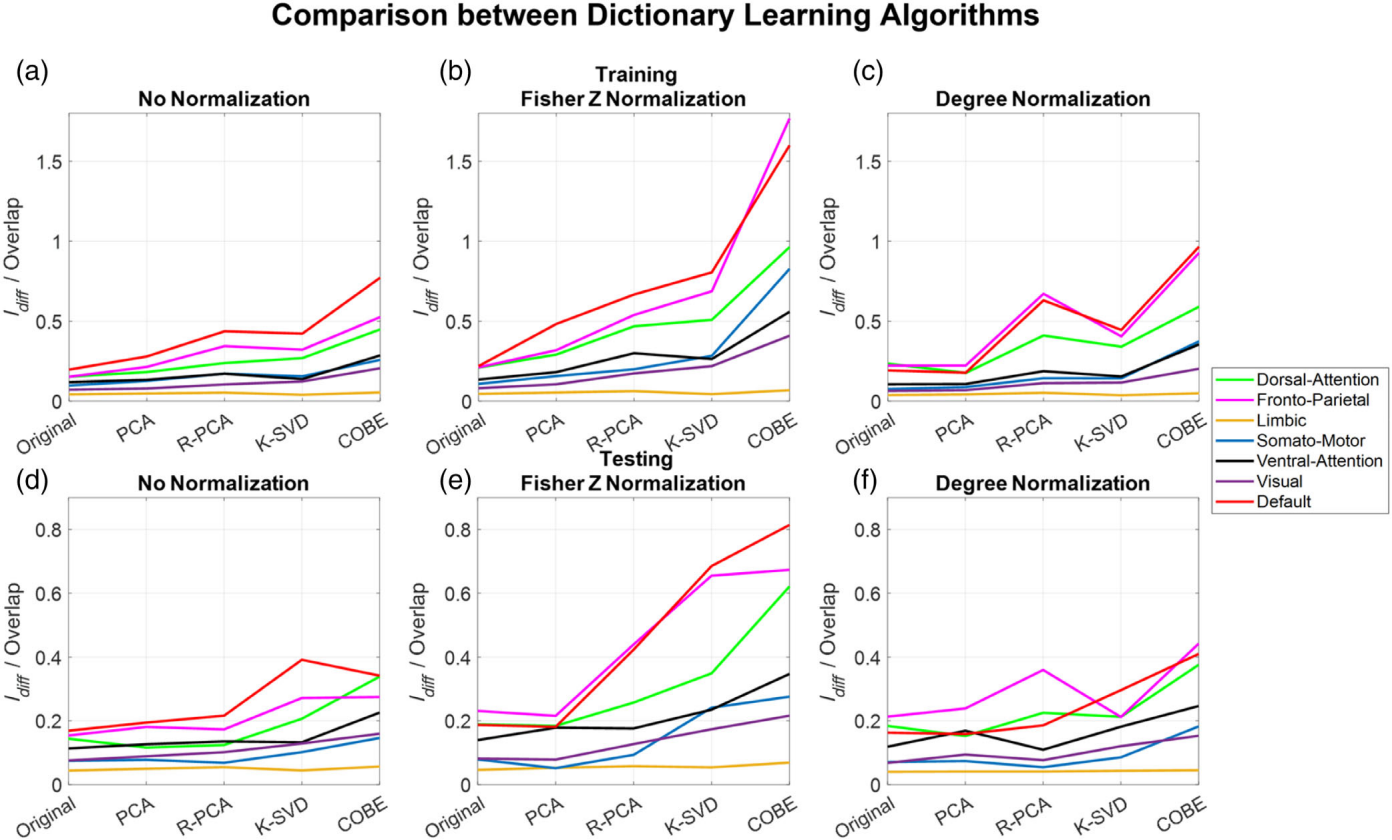
(a–c) Comparison between the dictionary learning algorithms during training. Following parameter check, we choose the parameters which maximize the ratio of $I_{\text{diff}}$ to Overlap and present the results here. (d–f) Comparison between the dictionary learning algorithms during test. Using the parameters and the thresholds for Overlap computation during training, these are the results obtained during the testing phase. Results were obtained using the Schaefer 400 atlas.

Here we compare the results obtained after choosing the best parameters that maximize the ratio of $I_{\text{diff}}$ to Overlap. Referring to Figure 11a–f, it is observed that the COBE algorithm with the Fisher Z transformation gave the best results with DMN during the training and testing phase by achieving the value of the ratio as high as 1.6 and 0.8, respectively. On the other hand, the LN, VN, and SMN seemed to have poor performance both during testing and training. The ratio for the LN remained unchanged, irrespective of the DL algorithms used. PCA, being computationally the simplest algorithm relative to the others mentioned here performed slightly better than the original raw correlations when none of the DL algorithms were used. RPCA performed exceptionally well when degree normalization was used. RPCA achieved a value of the ratio of around 0.7 for the DMN and FPN networks during training which is better than KSVD which achieved a value around 0.5. Using no normalization or Fisher Z normalization KSVD performs better than RPCA.

### 3.4 Variation with scan length

**FIGURE 12:**
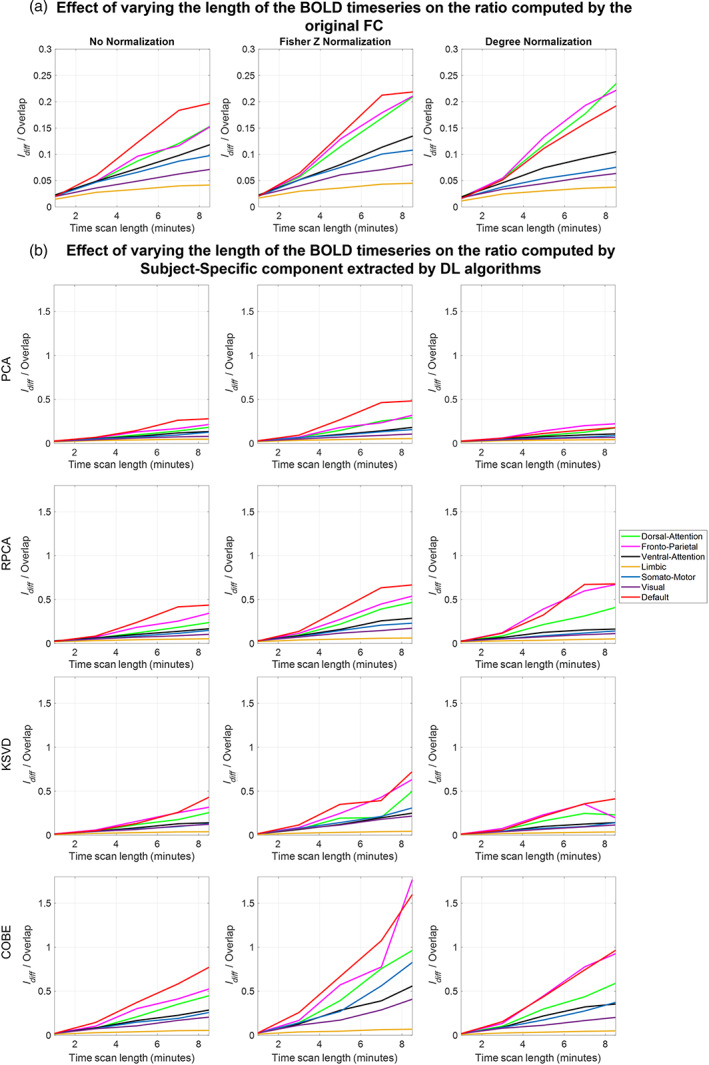
Effect of varying the number of time points in the BOLD signal on the ratio of $I_{\text{diff}}$ to Overlap computed using the (a) the original functional connectivity (b) the subject‐specific functional connectivity extracted by DL algorithms during the training phase. The time points were varied as 1, 3, 5, 7, and 8.5 min. Results were obtained using the Schaefer 400 atlas.

Figure 12 shows the effect of variation of BOLD time points on the ratio of $I_{\text{diff}}$ to Overlap computed using the original FC and the subject‐specific FC extracted from the 4 DL algorithms during the training phase (Figure S5 shows the results obtained during the testing phase). We observed that during both training and testing phases the ratio increased as the number of time points increased, suggesting that FC computed using more time points give better subject‐specific components. However, LN does not show much variation as the time scan length increased. Referring to Figure 12, the ratio remained constant at around 0.05 for LN. The ratio for DMN, FPN, and the DAN networks increased as more and more time points are used to compute the FC, suggesting more potential in these networks to enhance the individual components, with the exception of the RPCA algorithm where the DMN network saturated after 7 min to value of 0.4, 0.7, and 0.7 with no normalization, Fisher Z normalization, and degree normalizations, respectively. DMN along with Fisher Z transform acquired the maximum value of the ratio among other networks. Before using the DL algorithms with fisher Z normalization DMN achieved a value of 0.2, with PCA the value increased to 0.5, RPCA and KSVD had similar values around 0.7, and with COBE the value was maximum at 1.6. In comparison, the values of DMN with no normalization were lower with COBE achieving the maximum at just 0.75. Fisher Z transformed FC extracted the best Individual components as compared to no normalized and degree normalized FC.

### 3.5 Variation with atlas

**FIGURE 13:**
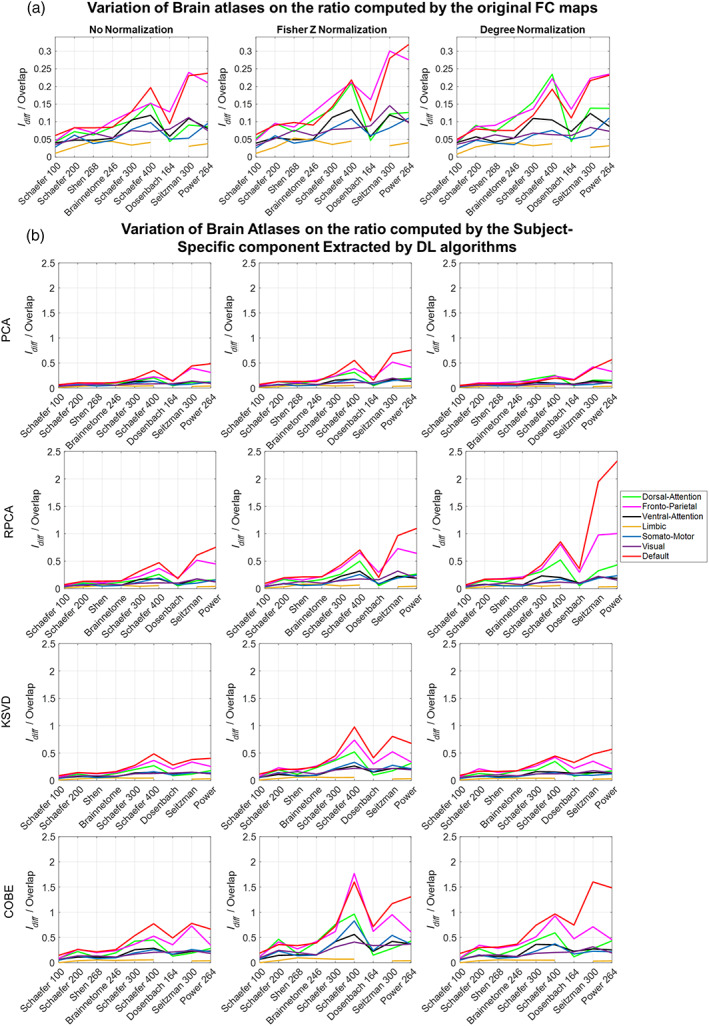
Effect of variation of brain atlas on the ratio of $I_{\text{diff}}$ to Overlap during the training phase. On the x‐axis brain atlas are arranged in decreasing order of average number of voxels per ROI.

Looking at Figure 13 we observed the effect of varying the brain atlas on the ratio of $I_{\text{diff}}$ to Overlap computed using the original FC and the subject‐specific FC extracted from the 4 DL algorithms during the training phase (refer Figure S6 for the results obtained during the testing phase). The Brain atlases are arranged in decreasing order of average number of voxels per ROI, with Schaefer 100 having the largest average number of voxels per ROI and power having the least (refer Table 1). Considering results obtained during the training and testing phases we observed that the less the average number of voxels per ROI in the brain atlas, the higher the ratio $I_{\text{diff}}/\text{Overlap}$. We also observed a dip at the Dosenbach atlas in both the training and testing phases across all the DL algorithms. Dosenbach atlas, although having a low average number of voxels per ROI, has only 164 ROI, which is very less as compared to the ones in its neighbor (Seitzman having 300 ROIs, and Schaefer 400 having 400 ROIs). This suggested that less Average number of voxels per ROI and a greater total number of ROIs are desirable.

### 3.6 Stability

**FIGURE 14:**
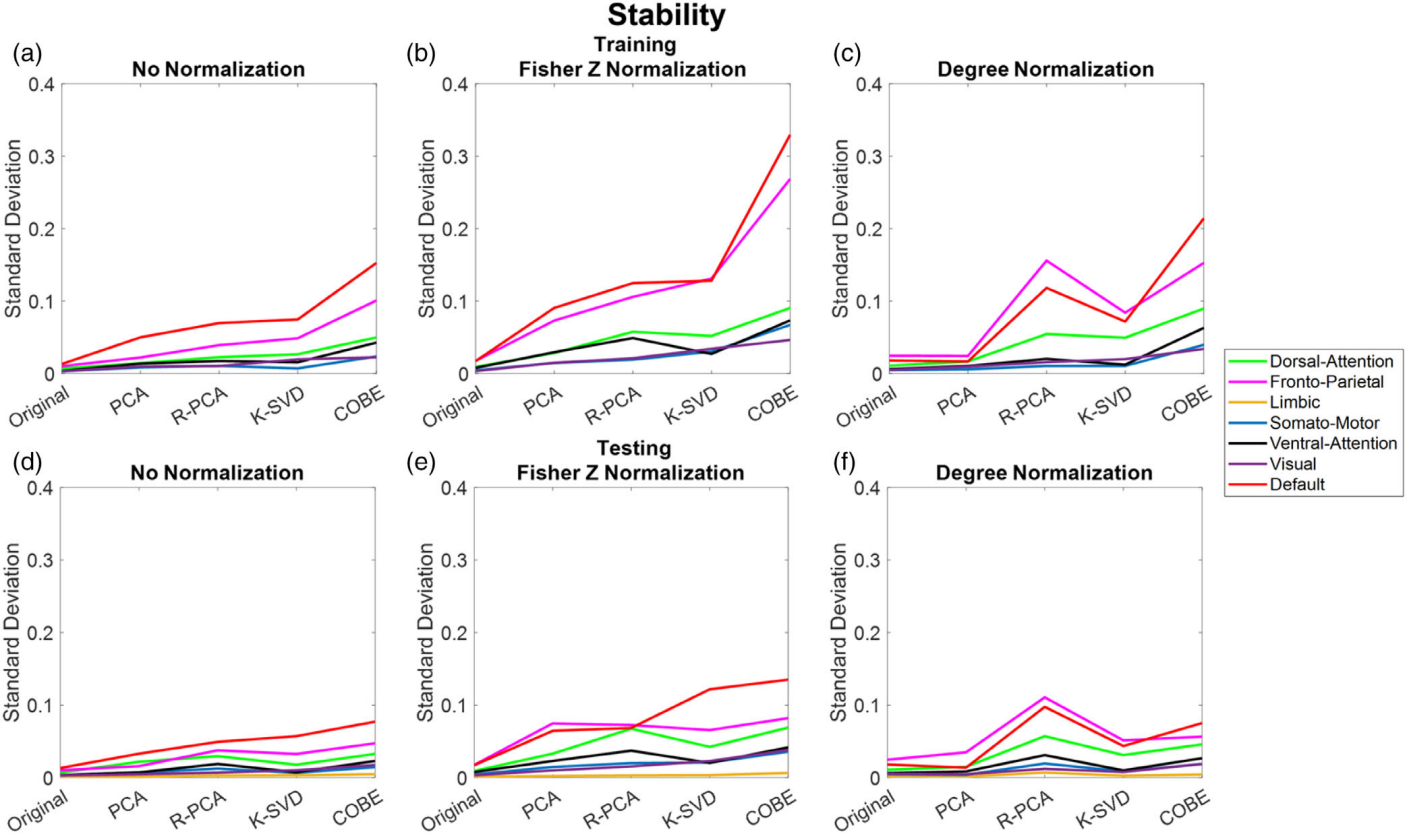
Results were repeated 252 $({}^{10}C_{5}$) times, each time taking a different set of training and testing data for the different dictionary learning algorithms. (a–c) The standard deviation of the ratio computed at each iteration across the different networks and the different normalization methods during training. (d–f) The results during the test.

The algorithms were repeated 252 $\left({}^{10}C_{5}\right)$ times (by choosing 5 scans out of the available 10 scans for training), each time picking a different combination of scans for training and testing to check the Algorithm's stability. Figure 14 shows the standard deviation of the ratio of $I_{\text{diff}}$ to Overlap found at each iteration across the networks and the normalization methods. Overall, the maximum standard deviation was around 0.3 for the DMN and FPN with Fisher Z transformation using the COBE algorithm, while others were primarily below 0.15 during training. After COBE, RPCA had the second‐largest standard deviation (around 0.15), especially with the degree normalization with DMN and FPN. DMN and FPN were observed to have a larger standard deviation as compared to the other networks. LN showed the least standard deviation across all the DL algorithms as well as the Normalization methods. KSVD had a lower standard deviation as compared to RPCA and COBE. Moreover, PCA results were the most stable with the least standard deviation, especially with the degree normalization. The more the standard deviation the less the stability as more standard deviation suggests that the results are dependent on which combination of subjects are used as training and testing. Ideally, the results should only be dependent on the DL algorithm, normalization method and the brain atlases used. Looking at Figure 14 we observed that despite COBE having a high standard deviation (especially for DMN and FPN using the Fisher Z normalization), the minimum value of the ratio of $I_{\text{diff}}$ to Overlap (which is 0.9 for DMN) is still higher than the maximum value of KSVD (which is 0.8 for DMN). Making COBE a better choice over KSVD. An important point to note is that the stability results were very similar between training and test which showed that all the algorithms have a good generalization.

### 3.7 Time taken by each algorithm

We also recorded the time taken by the algorithm to compute the subject‐specific FC to compare their performance with respect to the amount of time they take. These results were recorded on a Linux mint machine with an intel core i7 processor and 16 GB RAM. Further, the MATLAB 2019b was used to compute all the results of this study. Figure S4 shows the time taken by the algorithms across Normalization methods as well as across networks using Schaefer 300 nodes atlas. PCA took the least amount of time, being the simplest algorithm among the others. Comparatively, RPCA is computationally more costly, as it first had to run the RPCA algorithm and then the ODL DL algorithm. Thus, it took the most amount of time. COBE and K‐SVD took almost a similar amount of time. Since the time taken by the algorithms depended on the dimensionality of the data matrix, which is the number of elements in the FC upper triangular vector ($\tilde{n}$), we observed that the different rs‐networks took different time. Default mode had maximum number of nodes and hence had the largest $\tilde{n}$ thus it took the most amount of time across all the DL algorithms and normalization methods.

## 4 DISCUSSION

In this study, we compared four dictionary algorithms, PCA, RPCA, KSVD, and COBE based on how accurately and efficiently they can identify individual components from the FC of 7 large‐scale RSNs. We observed the COBE algorithm to work better than the other DL algorithms when the Fisher Z transform was used. DMN and FPN networks performed better than the other networks. The FC computation is largely dependent on the Brain Atlas. The spatial extent of each ROI used for seed location as well as the total number of ROIs affects the FC. In this study, we have considered both these aspects. We observe the variation of the ratio of $I_{\text{diff}}$ to Overlap across 10 different atlas having nodes varying from 100 to 400. We also highlighted the average number of voxels per ROI which tells us about the average ROI size in these atlases. Our results indicate that the more granular ROIs, that is, lesser the average number of voxels contained per ROI, the better the ratio of $I_{\text{diff}}$ to Overlap and hence the more accurate extraction of Subject‐Specific components. We average the time series of the voxels in an ROI to get one single time series for that ROI. If there are more voxels per ROI, then we may lose information as the mean time series may not be a good representation of the region. However, if we look at Figure 13 a dip in the ratio can be observed for the Dosenbach atlas. Dosenbach Atlas has a relatively low number of average voxels per ROI and a significantly lower total number of ROIs (164), which may be responsible for the dip. This dip suggests that averaging a lower number of voxels per ROI and a greater number of ROIs is desirable for obtaining a more accurate estimate of the subject‐specific FC. An ideal case would be to do the analyses at the voxel level, where each voxel would be considered a node. However, computing the FC, in this case, would significantly increase the computational complexity as the voxels in the brain are in the order of $10^{5}$. After calculating the FC, the order may go up to $10^{10}$, which would make it very difficult and time‐consuming for the DL algorithms to learn the Dictionaries. Furthermore, to deal with such high dimensional data, we would also require more subjects, the number of subjects used should be comparable to the dimensions for the DL algorithm to work. Also, massively univariate approaches like these make a very strong assumption about independence between voxels, which is in direct contradiction to the ubiquitous regional homogeneity among brain regions. If the multiplicity issue is not addressed properly, we may wrongly model a true effect.

Large‐scale RSNs are generally associated with specific cognitive functions as pointed out by Hausman et al. (2020), such as attention (Fox et al., 2006), memory (Vincent et al., 2006), cognitive control (Cole et al., 2012; Dosenbach et al., 2007; Vincent et al., 2008), default mode (Buckner et al., 2008; Raichle et al., 2001), motor (Biswal et al., 1995), and sensory systems (Damoiseaux et al., 2006; De Luca et al., 2005). By incorporating a set of DL algorithms, this study focuses on determining which of the RSNs from Yeo et al. (2011) can be used to estimate the individual variability in a subject. Our results show that DMN and FPN networks demonstrate higher individual variability in FC compared to other RSNs. However, it is essential to note that in this study the atlases used for the computation of the RSFC have more nodes in DMN than FPN, which affects the performance of the DL algorithm. Yet, FPN does equally good as DMN in most cases. Studies in literature have pointed out that the FPN is potentially a better candidate to extract the subject‐specific component (Amico & Goñi, 2018; Cai et al., 2020). On the other hand, LN has performed poorly across all DL algorithms and atlases. There may be three reasons for this (1) LN may not have a significant subject‐specific component, (2) As limbic nodes are physically below the other networks, the signal from the limbic region could be noisy, or (3) The number of nodes in LN is lesser as compared to the other networks, which have a great influence on the learned dictionaries. VN and SMN also performed poorly, which is expected, as, in resting‐state, all the subjects lie still in the scanner without doing any motor or visual activity. It would be interesting to recheck the results of these networks with a task comprising of a visual or motor activity.

Duration of the fMRI scan can be a critical contributing factor, given the fact that being in the MRI scanner for an extended period can be difficult for patients. Thus, we also looked at the effect of varying the time points on the ratio of $I_{\text{diff}}$ to Overlap (refer Figure 12 and Figure S5). Results suggest that a longer duration can efficiently bring out better Individual connectome only in specific RSNs such as default mode, fronto‐parietal, and dorsal attention. However, other networks tend to saturate after at most 5 min of scan length, except the default mode network enhanced by the RPCA algorithm. Studies like Amico and Goñi (2018) have also pointed out similar results but considering the whole brain and not the RSNs.

In this study, we compared four dictionary algorithms, (i) PCA, (ii) RPCA, (iii) KSVD, and (iv) COBE algorithms, respectively. COBE algorithm has achieved the best performance in all the results mentioned in this study; however, it is essential to note that it is supervised. A subject matrix $Y_{i}$ is given as input to the COBE algorithm during the training phase, which means the COBE algorithm knows which scans belong to the same subject. This could be the primary reason behind its improved performance. On the contrary, COBE demonstrated stability which is lesser than the other algorithms mentioned (refer to Figure 14), suggesting that COBE may be prone to overfitting as compared to other algorithms. KSVD, on the other hand, has a better performance as compared to PCA and performs as good as RPCA. KSVD has better stability as compared to COBE and RPCA. RPCA does an excellent job with degree normalized FC, especially with the DMN, results are better than KSVD and close to COBE but on the cost of reduced stability. Also, RPCA requires a substantial amount of time because it consists of two sequentially costly algorithms—RPCA and ODL. Despite that, we only required this time during the training phase. All the algorithms may take almost the same amount of time in the testing phase, as a least‐squares solution or simple matrix multiplication must be computed from the learned dictionaries. Furthermore, PCA, the most primitive algorithm, takes the least amount of time for all the networks and is the most stable. However, its performance is just slightly better as compared to when no DL algorithm was applied and the FC was used directly, but not as good as the other algorithms as far as the ratio of $I_{\text{diff}}$ to Overlap is considered.

Based on our evaluation on the type of normalization applied, choice of normalization does play a significant role in enhancing the individual‐specific components. Although Degree normalization was observed to be better as compared to the Fisher's Z transform before using any DL algorithm (refer to Figure S2), Fisher's Z transform generates more accurate estimates after applying the DL algorithms, except for RPCA. Therefore, an evaluation of different normalization techniques may be necessary before using any new method, and one may then choose the best among them.

## 5 CONCLUSIONS

We systematically reviewed and compared four DL algorithms stating their time complexity and reproducibility using the network‐specific FC to get the individual specific components. We found that the COBE algorithm with default‐mode and fronto‐parietal networks performed comparatively better than other RSNs, while LN, VN, and SMN networks performed poorly for all DL algorithms. We also studied the effect of different normalization methods and observed Fisher Z transform to be better than the degree and no normalization. Finally, we computed the results using nine distinct Brain Atlases of different resolutions and affirmed that a greater number of ROIs per atlas and a lesser average number of voxels per ROIs achieve better results.

## Supporting information


**Data S1.** Supporting Information.

## Data Availability

The MSC dataset is publicly available at https://openneuro.org/datasets/ds000224, and the HNU dataset is publicly available at the Consortium for Reliability and Reproducibility database at http://fcon\_1000.projects.nitrc.org/indi/CoRR/html/hnu\_1.html Data were processed using publicly available software (SPM12 http://www.fil.ion.ucl.ac.uk/spm/software/spm12/). The Subject Specific components were extracted by code written in MATLAB. The code is available from the authors upon request.
