# Supplementary material for: Enhancing the network specific individual characteristics in rs‐fMRI functional connectivity by dictionary learning
Source: Hum Brain Mapp. 2023 Apr 18;44(8):3410–32. doi: 10.1002/hbm.26289 (PMC10171559; doi:10.1002/hbm.26289)
Supplement: Supplementary file 1 — Data S1. Supporting Information. [file HBM-44-3410-s001.docx]

**Supplementary document**

Enhancing the Network Specific Individual Characteristics in rs-fMRI Functional Connectivity by Dictionary Learning

**Degree Normalization**

Figure S1 shows the steps involved in the computation of Degree Normalization.


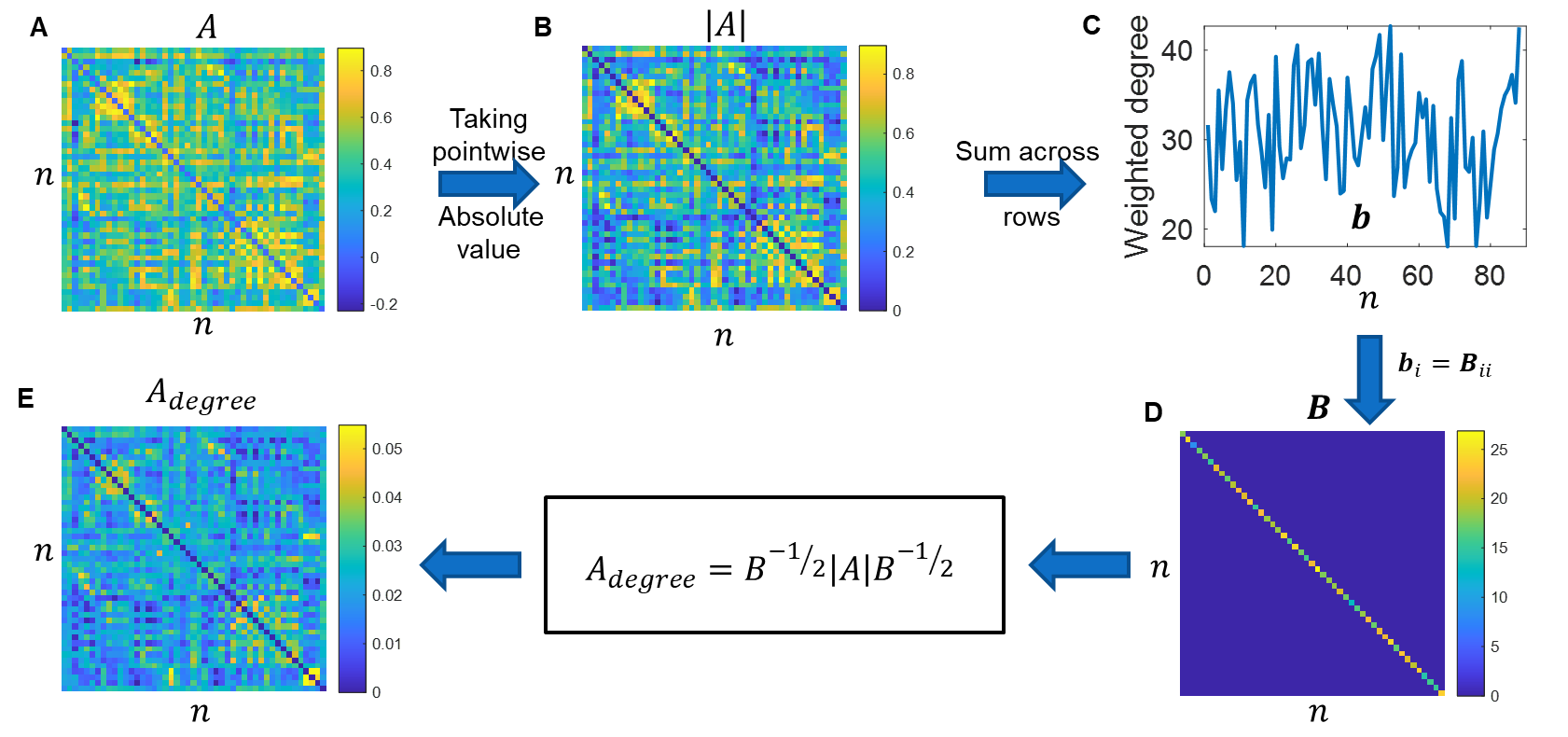


Figure S1 Degree Normalization: (A) FC matrix $A$ computed by Pearson correlation between BOLD signals of a rs-network. The range of values lie in between [-1 1]. (B) Taking absolute values of every element, now the range becomes [0 1]. (C) Taking summation across rows of $|A|$ matrix. (D) Degree Matrix $\boldsymbol{B}$ is obtained by putting the weighted degree $b$ on the diagonal and zeros elsewhere. (E) Degree Normalized matrix.

**Ratio of** $\boldsymbol{I}_{\boldsymbol{diff}}$ **to** $\boldsymbol{Overlap}$ **before applying any Algorithm**

Figure S2 shows the variation of the ratio of $I_{diff}$ to $Overlap$*,* when computed over the subjects’ FC maps directly before using any DL algorithms.

**
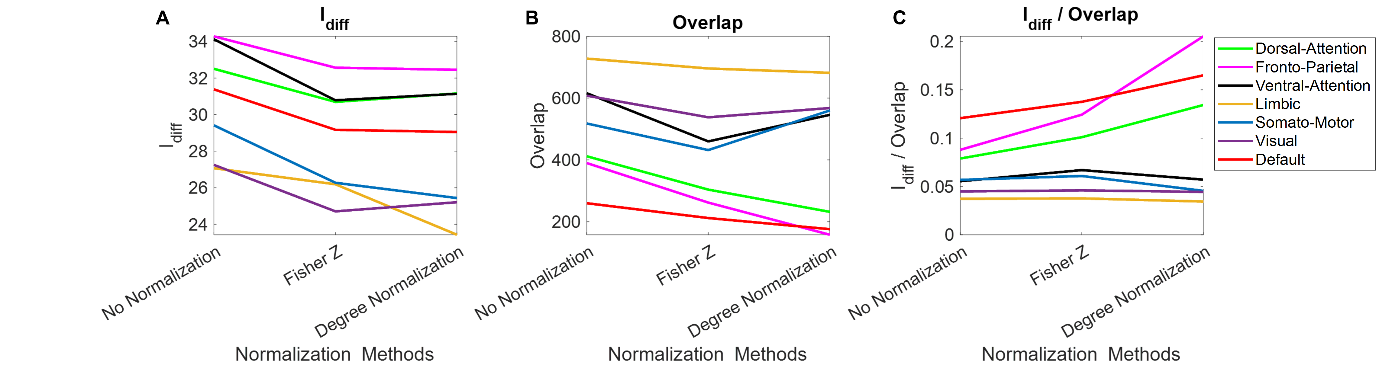
**

Figure S2 (A-C) $I_{diff}$, $Overlap$ and Ratio of $I_{diff}$ to $Overlap$ computed over the FC matrix directly before using any DL algorithms across different Normalization methods on the x-axis as well as different Resting State Networks represented with colours.

**Percentage of variance explained by the PCA components**

Figure S3 shows the percentage of variance explained by the PCA components across the normalization methods and the resting state networks.


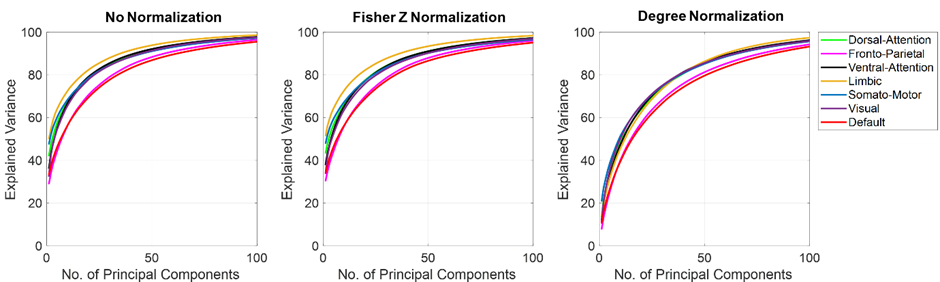


Figure S3 Percentage of variance explained by the PCA components.

**Time Taken by each DL algorithm.**

Figure S4 demonstrates the time taken to train the dictionaries of each of the DL algorithms. These results were recorded on a Linux mint machine with an intel core i7 processor and 16 GB RAM. Further, MATLAB 2019b was used to compute all the results of this study.


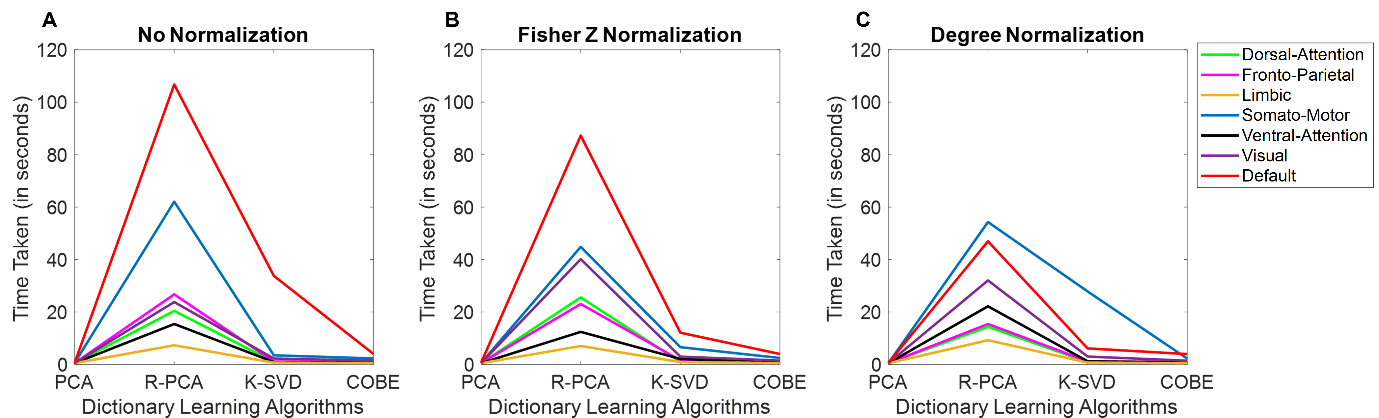


Figure S4 Time taken by DL algorithms: Plot shows time taken by the DL algorithms across all rs-networks and Normalization methods (A)-(C), using Schaefer 300 Atlas.

**Effect of varying the length of BOLD timeseries on the ratio computed by Subject-Specific component extracted by DL algorithms during test**

**
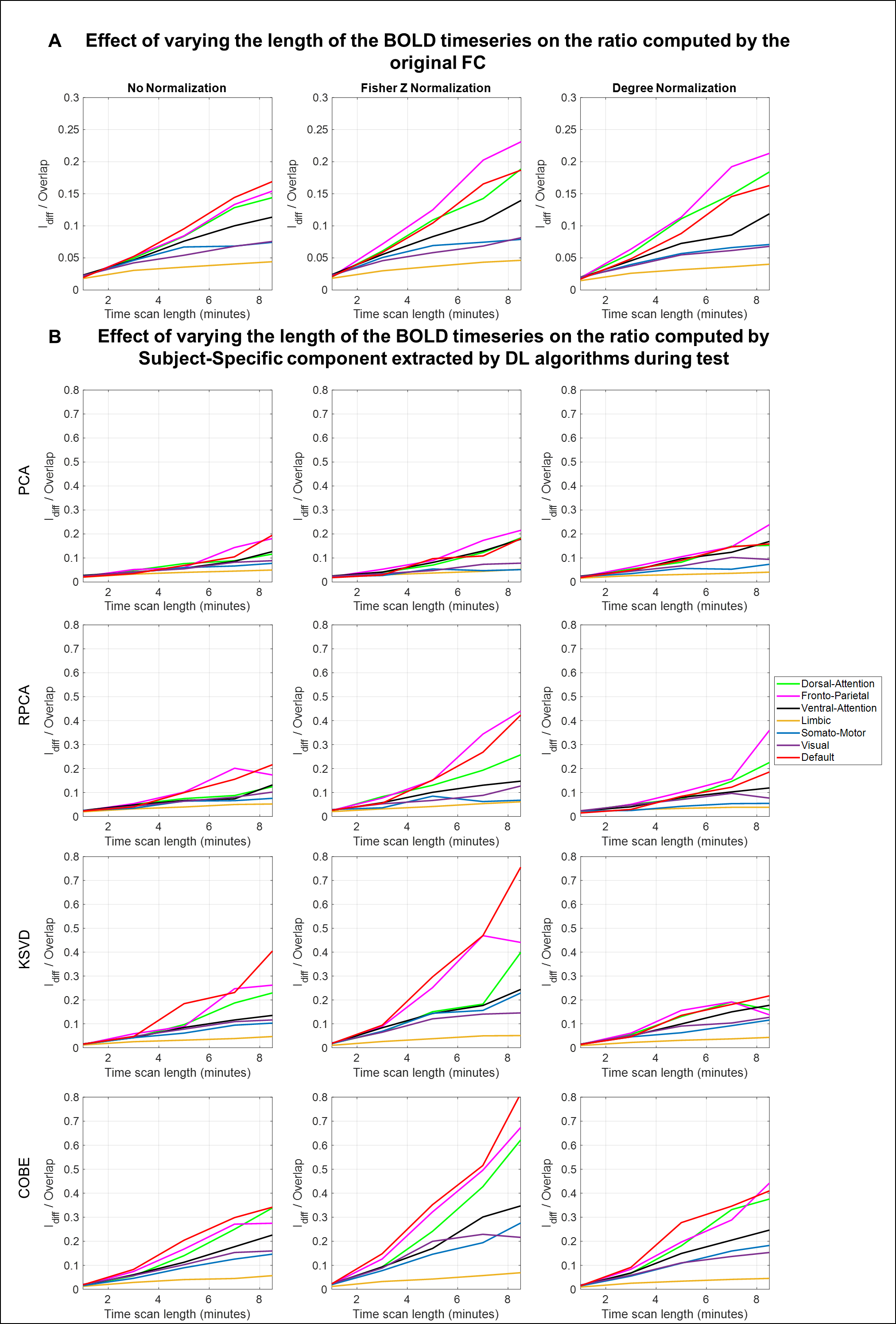
** Figure S*5* : Effect of varying the no. of time points in the BOLD signal on the ratio of $I_{diff}$ to $Overlap$ computed using the (**A**) the original Functional Connectivity (**B**) the Subject-Specific Functional Connectivity extracted by DL algorithms during the training phase. The time points were varied as (1,3,5,7,8.5) minutes. Results were obtained using the Schaefer 400 atlas.

**Effect of variation of Brain Atlases on the ratio computed by the Subject-Specific component Extracted by DL algorithms during test**

**
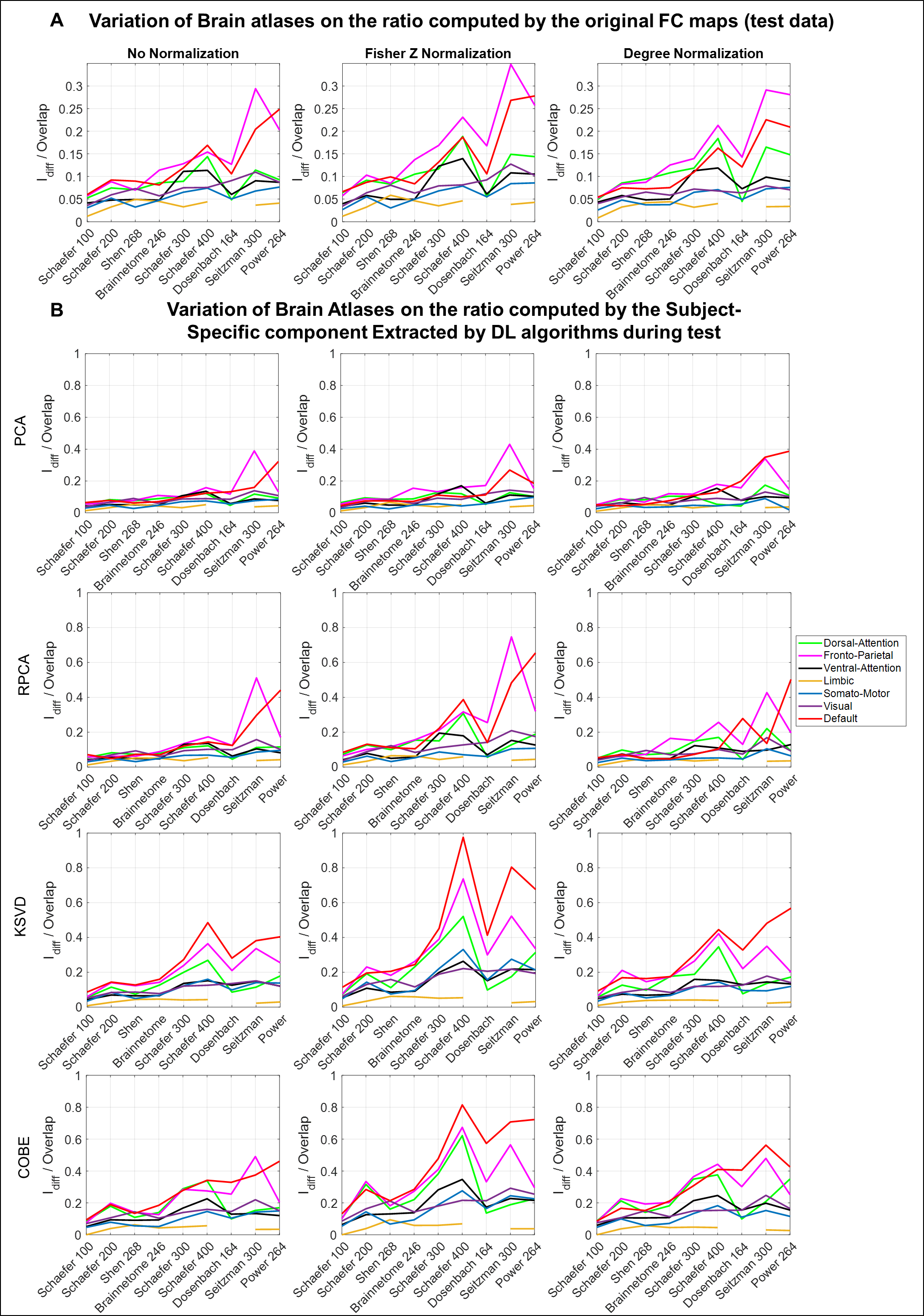
** Figure S6: Effect of variation of Brain Atlas on the ratio of $I_{diff}$ to $Overlap$ during the training phase. On the x-axis Brain Atlas are arranged in decreasing order of average no. of voxels per ROI.

**No. of Nodes in 7 rs-networks across Atlas**

Figure S5 demonstrates the number of nodes present in each of the 7 resting-state networks for every atlas used in the study. We observed that, overall DMN has the maximum number of nodes (except Dosenbach atlas) and LN has the least number of nodes (except Shen atlas) for most of the atlases.

**
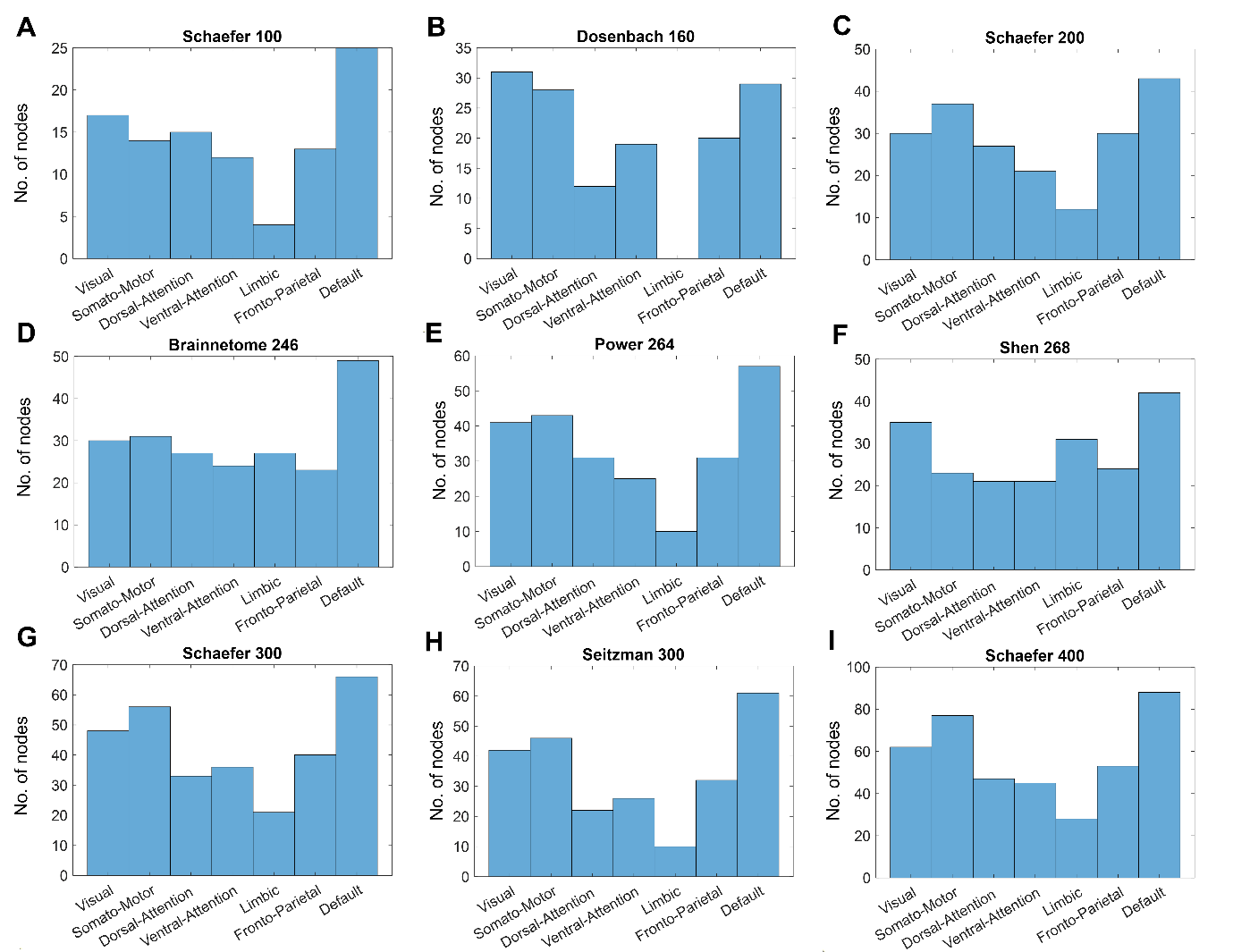
**

Figure S5 No. of Nodes in the 7 Yeo rs-networks present in all the different atlases mentioned in this study


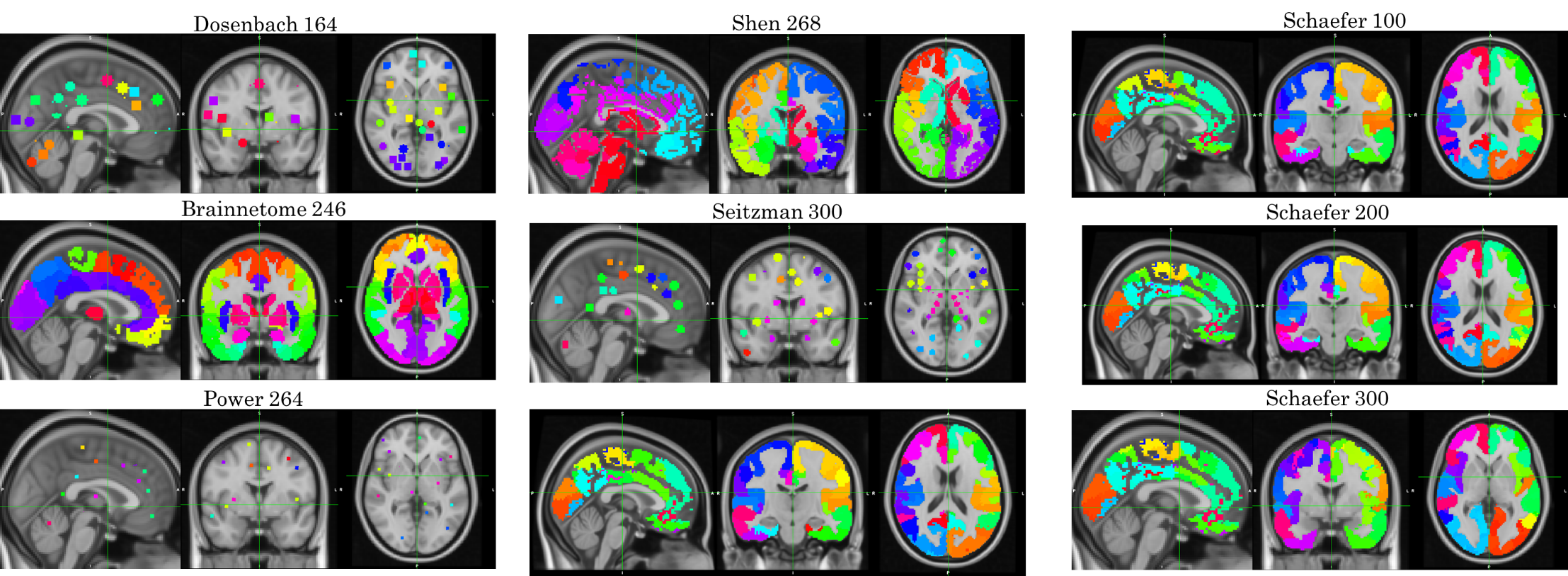


Figure S6 Brain atlases used in this study.

Figure S6 shows the brain atlases used to compute the FC in this study. All the atlases used in this study at in the 2mm MNI space. Dosenbach, Power and Seitzman atlases have spherical ROIs where not every voxel in the brain is assigned to a region. Thus these atlases have less voxels per region inspite of having a large total number of regions
